# Hypoxia‐Induced FUS–circTBC1D14 Stress Granules Promote Autophagy in TNBC

**DOI:** 10.1002/advs.202204988

**Published:** 2023-02-19

**Authors:** Ying Liu, Yiwei Liu, Yinqiao He, Ning Zhang, Siyue Zhang, Yaming Li, Xiaolong Wang, Yiran Liang, Xi Chen, Weijing Zhao, Bing Chen, Lijuan Wang, Dan Luo, Qifeng Yang

**Affiliations:** ^1^ Department of Breast Surgery General Surgery Qilu Hospital of Shandong University Ji'nan Shandong 250012 P. R. China; ^2^ Pathology Tissue Bank Qilu Hospital of Shandong University Ji'nan Shandong 250012 P. R. China; ^3^ Research Institute of Breast Cancer Shandong University Ji'nan Shandong 250012 P. R. China

**Keywords:** autophagy, circTBC1D14, fused in sarcoma (FUS), hypoxia, stress granule

## Abstract

Triple‐negative breast cancer (TNBC) is a highly aggressive subtype of breast cancer that is suggested to be associated with hypoxia. This study is the first to identify a novel circular RNA (circRNA), circTBC1D14, whose expression is significantly upregulated in TNBC. The authors confirm that high circTBC1D14 expression is associated with a poor prognosis in patients with breast cancer. circTBC1D14‐associated mass spectrometry and RNA‐binding protein‐related bioinformatics strategies indicate that FUS can interact with circTBC1D14, which can bind to the downstream flanking sequence of circTBC1D14 to induce cyclization. FUS is an essential biomarker associated with stress granules (SGs), and the authors find that hypoxic conditions can induce FUS–circTBC1D14‐associated SG formation in the cytoplasm after modification by protein PRMT1. Subsequently, circTBC1D14 increases the stability of PRMT1 by inhibiting its K48‐regulated polyubiquitination, leading to the upregulation of PRMT1 expression. In addition, FUS–circTBC1D14 SGs can initiate a cascade of SG‐linked proteins to recognize and control the elimination of SGs by recruiting LAMP1 and enhancing lysosome‐associated autophagy flux, thus contributing to the maintenance of cellular homeostasis and promoting tumor progression in TNBC. Overall, these findings reveal that circTBC1D14 is a potential prognostic indicator that can serve as a therapeutic target for TNBC treatment.

## Graphical abstract

FUS‐circTBC1D14‐associated stress granules (SGs) are transferred to the cytoplasm after being modified by PRMT1 and circTBC1D14 increases the stability of PRMT1 by inhibiting its K48‐regulated polyubiquitination. Then FUS‐circTBC1D14 can initiate a cascade of SGs‐linked proteins to control the elimination of SGs by recruiting LAMP1. The findings reveal that circTBC1D14 can serve as a therapeutic target for TNBC treatment.

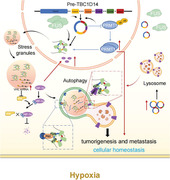

## 1 Introduction

Breast cancer has exceeded lung cancer and is now the leading cause of cancer‐related mortality in women worldwide, with a 0.5% annual increase in incidence rate. ^[^ ^1^ ^]^ Triple‐negative breast cancer (TNBC) is the most crucial subtype of breast cancer, exhibiting the highest recurrence, metastasis, and mortality rates ^[^ ^2^ ^]^ and accounting for ≈15% of all breast cancer cases.^[^ ^3^ ^]^ TNBC is an aggressive heterogeneous malignancy subtype of breast cancer that lacks effective targeted strategies, and the hypoxic microenvironment of TNBC solid tumors poses a major treatment challenge.^[^ ^4^ ^]^ Therefore, it is necessary to identify the underlying molecular mechanisms to develop novel therapeutic agents for patients with TNBC.

Circular RNAs (circRNAs) contribute significantly to various human cancers, including TNBC,^[^ ^5^ ^]^ and the characterization of circRNA expression patterns has been described in different subtypes of breast cancer.^[^ ^6^ ^]^ circRNAs may contribute to tumor subtype distinction and can be used as novel diagnostic and prognostic biomarkers. circRNAs exert their critical biological roles as miRNA sponges, modulating gene transcription, affecting linear splicing, combing RNA‐binding proteins (RBPs), and modulating protein translation.^[^ ^7^ ^]^ For example, circRNA‐fork head box O3 (circFoxo3) prevents MDM2 from degrading Foxo3, thereby increasing the Foxo protein levels and decreasing the p53 levels in TNBC.^[^ ^8^ ^]^ In addition, circRNA‐roundabout guidance receptor 1 (circROBO1) interacts with fused in sarcoma (FUS), promoting the back splicing of circRNAs.^[^ ^9^ ^]^ circRNA‐mitochondrial tRNA translation optimization 1 interacts with the TNF receptor‐associated factor 4, which is suppressed by the competing endogenous RNA (ceRNA) molecular mechanism and activates Eg5 translation to modulate TNBC resistance to monastrol.^[^ ^10^ ^]^ Therefore, RBP‐dependent circulation and circRNA–RBP interactions play essential roles in the occurrence and progression of TNBC.

In the present study, we characterized a circRNA, termed circTBC1D14, derived from the back‐splicing of exons 2–7 of *TBC1D14*, whose expression levels were upregulated in TNBC tissue samples and correlated with poor survival.

We discovered that hypoxia‐induced protein arginine methyltransferase 1 (PRMT1) could regulate the transfer of FUS–circTBC1D14 to the cytoplasm, thus promoting stress granule (SG) formation in TNBC cells. circTBC1D14 suppressed the ubiquitination of PRMT1 protein, thereby increasing the tumor aggressiveness of TNBC. Furthermore, FUS–circTBC1D14 SGs in the cytoplasm could induce autophagic flux by recruiting the lysosomal‐associated membrane protein 1 (LAMP1) under hypoxic conditions. Our findings reveal the significant role of circTBC1D14 in TNBC progression and suggest that it may serve as a diagnostic target for TNBC and other hypoxia‐associated tumors.

## 2 Results

### 2.1 circTBC1D14 Expression Levels are Significantly Upregulated in TNBC and Correlate with the Poor Prognosis of Patients

**Figure 1:**
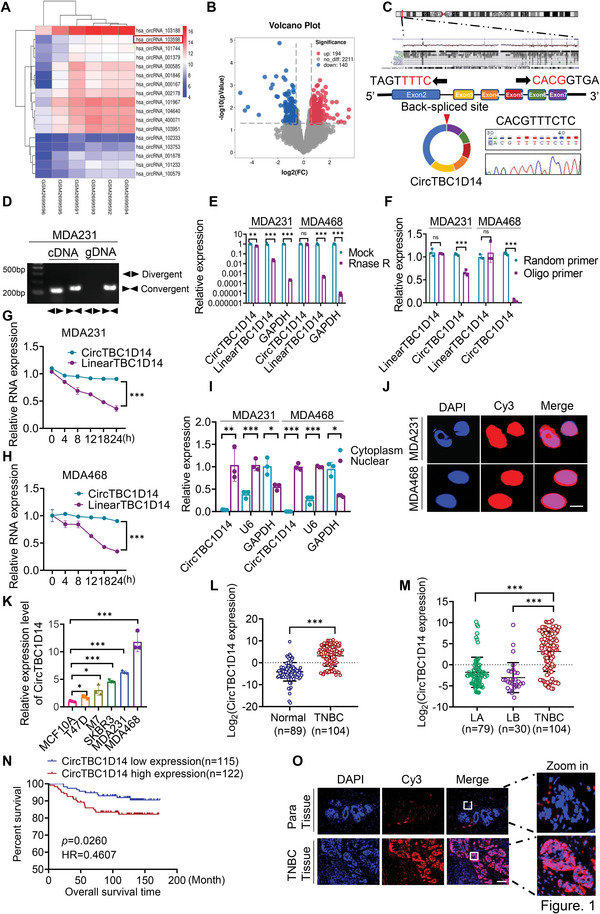
circTBC1D14 was significantly upregulated in TNBCs and contributed to the poor prognosis of patients. A) The circRNAs cluster heat map of TNBC tumor tissues and normal breast tissues. The red and blue strips indicated upregulated and downregulated circRNAs. B) The volcano plot of circRNAs expression profiles. C) Schematic diagram of circTBC1D14 formation by the circulation of exon2 to exon7 in TBC1D14 genes. The back‐spliced junction sequences of circTBC1D14 were validated by Sanger sequencing. D) Gel electrophoresis analysis to detect the existence of circTBC1D14 and TBC1D14 from gDNA and cDNA in MDA231 and MDA468 cells respectively. E) RT‐qPCR of total RNA with or without RNase R treatment in MDA231 and MDA468 cells with convergent and divergent primers. two‐tailed unpaired t‐test. F) Reverse transcription experiments with random hexamer or oligo dT primers in MDA231 and MDA468 cells. Two‐tailed unpaired t‐test. G,H) RT‐qPCR assay with Actinomycin D treatments of circTBC1D14 and linear TBC1D14 in MDA231 and MDA468 cells. Two‐tailed unpaired t‐test. I) RT‐qPCR of the relative distribution of circTBC1D14 in MDA231 and MDA468 cells. U6 served as the nuclear RNA marker and GAPDH served as the cytoplasmic RNA marker. Two‐tailed unpaired t‐test. J) RNA fluorescence in situ hybridization (FISH) of circTBC1D14 in MDA231 and MDA468 cells. The red region showed the distribution of the circTBC1D14 probe, and the blue region showed the nuclei staining by DAPI. Scale bar, 10 µm. K) RT‐qPCR analysis of circTBC1D14 expression in different breast cancer cells. two‐tailed unpaired t‐test. L) RT‐qPCR analysis of circTBC1D14 in normal tissues (*n* = 89) and TNBC tissues (*n* = 104) from Qilu hospital. Two‐tailed unpaired t‐test. M) RT‐qPCR analysis of circTBC1D14 in luminal A tissues (*n* = 79), luminal B tissues (*n* = 30), and TNBC tissues (*n* = 104) from Qilu hospital. Two‐tailed unpaired t‐test. N) Kaplan–Meier survival analysis of breast cancer patients from Qilu hospital according to the expression level of circTBC1D14 using the log‐rank test (*n* = 237). O) RNA FISH assays in human paired TNBC tissues and para tissues. The red region showed the distribution of the circTBC1D14 probe, and the blue region showed the nuclei staining by DAPI. Scale bar, 10 µm. The data are shown as the mean ± SD, NS (no significance) *p < 0.05 **p < 0.01, ***p < 0.001.

To identify the circRNAs responsible for tumor progression in TNBC, we analyzed TNBC tumor and normal breast tissue samples from the GSE101124 dataset on the Gene Expression Omnibus (GEO) database. With fold‐change > 1.0 and *t*‐test p < 0.05 as the threshold criteria, 194 upregulated and 140 downregulated circRNAs were identified. We found 17 differentially expressed circRNAs in the upregulated group with fold‐change > 2.5 and *t*‐test *p* < 0.05 (**Figure** **1**A,B). RT‐qPCR analysis of paired TNBC tissues and TNBC cell lines indicated that hsa_circRNA_103598 (circTBC1D14) was superior to hsa_circRNA_103188, which ultimately determined as target circRNA (Figure S1A,B, Supporting Information). We found that circTBC1D14 is formed by the circularization of exons 2–7 of *TBC1D14* using the University of California Santa Cruz Genome Browser (http://genome.ucsc.edu/), and Sanger sequencing also detected the back‐spliced junction site of exons 2 and 7 of circTBC1D14 in MDA231 cells (Figure 1C). To demonstrate the characteristics of circTBC1D14 in TNBC cells, divergent and convergent primers were designed to amplify circTBC1D14 and linear TBC1D14 transcripts, respectively. Polymerase chain reaction (PCR) revealed that circTBC1D14 was detected only in cDNA using divergent primers, but linear TBC1D14 transcripts were detected in both cDNAs and genomic DNA using convergent primers, which demonstrated that circTBC1D14 was a back‐spliced transcript rather than a trans‐spliced transcript (Figure 1D). We found that circTBC1D14 was more resistant to RNase R degradation than linear TBC1D14 transcripts (Figure 1E). However, when both oligo dT primers and random hexamers were used for RNA reverse transcription, linear TBC1D14 transcripts were detected in both random hexamers and oligo dT primers, while circTBC1D14 nearly disappeared with oligo dT primers (Figure 1F). We further evaluated the stability of circTBC1D14 using the actinomycin D assay. The results showed that the half‐life of circTBC1D14 was longer than that of linear circTBC1D14 (Figure 1G,H), which demonstrated that circTBC1D14 was more stable in MDA231 and MDA468 cells. To explore the subcellular localization of circTBC1D14, we performed nuclear and cytoplasmic extraction assays in TNBC cells, and reverse transcription‐quantitative polymerase chain reaction (RT‐qPCR) analysis showed that circTBC1D14 was mostly located in the nucleus rather than in the cytoplasm (Figure 1I). Fluorescence in situ hybridization (FISH) assays revealed that circTBC1D14 was primarily located in the nucleus (Figure 1J). To determine the expression levels of circTBC1D14 in breast cancer cells, we performed RT‐qPCR and found that circTBC1D14 levels were upregulated in MDA231 and MDA468 TNBC cell lines (Figure 1K). RT‐qPCR analysis of breast cancer and adjacent normal breast tissues revealed that circTBC1D14 levels were upregulated in breast cancer samples (Figure 1L). Compared to the luminal A/B subtype of breast cancer, circTBC1D14 was enriched in TNBC (Figure 1M). Simultaneously, Kaplan–Meier analysis revealed that high expression of circTBC1D14 predicted poor overall survival (Figure 1N). RNA FISH assays using TNBC and adjacent normal breast tissues revealed that circTBC1D14 levels were significantly upregulated in TNBC tissues than in the adjacent normal breast tissues (Figure 1O). These results confirmed that circTBC1D14 is highly expressed in TNBC cells and tissues and may contribute to TNBC progression.

We investigated the relationship between circTBC1D14 expression and the clinicopathological characteristics of patients with TNBC. The results showed that high expression of circTBC1D14 was positively correlated with large tumor size, Ki67 expression, lymph node metastasis, and distant metastasis, while there was no significant correlation between circTBC1D14 and patient age or histological grade (**Table** **1**). In addition, we conducted univariate and multivariate Cox proportional hazards regression analyses to evaluate the prognostic factors for patients with TNBC and found that circTBC1D14 could be considered as an independent prognostic factor for the overall survival of patients (**Table** **2**). Together, these data demonstrate that circTBC1D14 plays an oncogenic role in TNBC.

**Table 1:** Association between clinicopathological variables and circTBC1D14 expression in patients with TNBC

	CircTBC1D14 expression		
Variable	LOW (*n* = 62)	HIGH (*n* = 63)	P
Age			
<50	30 (48.39)	37 (58.73)	0.411
≥50	32 (51.61)	26 (41.27)	
Tumor size			
<T1	43 (69.35)	30 (47.62)	**0.003**
≥T1	19 (30.65)	33 (52.38)	
Grade			
G1/G2	38 (61.3)	29 (46.03)	0.127
G3	24 (38.7)	34 (53.97)	
KI67			
<40	36 (58.06)	24 (38.1)	**0.025**
≥40	26 (41.94)	39 (61.9)	
LN metastasis			
0	39 (62.9)	31 (49.21)	**0.036**
1‐3	18 (29.03)	15 (23.81)	
3	5 (8.07)	17 (26.98)	
Distant metastasis			
N	58 (93.55)	49 (77.78)	**0.012**
Y	4 (6.45)	14 (22.22)	

Patients were divided into circTBC1D14 low (*n* = 62) and circTBC1D14 high (*n* = 63) groups. Clinical variables included patient age, tumor size, histological grade, Ki‐67, LN (lymph node) metastasis, and distant metastasis. *p* values were determined via a two‐tailed chi‐square test.

**Table 2:** Univariate and multivariate analyses of prognostic factors for patients with TNBC

	Univariate analysis		Multivariate analysis	
Variable	HR (95% CI)	P	HR (95% CI)	P
Age				
<50	Reference			
≥50	0.377 (0.104‐1.373)	0.139		
Tumor size				
<T1	Reference			
≥T1	2.327 (0.771‐7.026)	0.134		
Grade				
G1/G2	Reference		Reference	
G3	2.429 (1.134‐5.204)	**0.022**	2.773 (1.126‐6.83)	**0.027**
KI67				
<40	Reference		Reference	
≥40	4.607 (1.018‐20.842)	**0.047**	3.203 (0.664‐15.441)	0.147
LN metastasis				
N	Reference		Reference	
Y	3.969 (1.071‐14.702)	**0.039**	2.651 (0.683‐10.282)	0.159
Distant metastasis				
N	Reference		Reference	
Y	67.179 (8.658‐521.244)	**≤0.001**	34.506 (3.993‐298.215)	**0.001**
CircTBC1D14 expression				
LOW	Reference		Reference	
HIGH	19.728 (2.534‐153.067)	**0.004**	27.392 (3.162‐237.272)	**0.003**

Variables included patient age, tumor size, histological grade, LN (lymph node) metastasis, distant metastasis, and the expression of Ki‐67, and circTBC1D14. Variables were analyzed using a univariate Cox regression model, and the *p* values were determined through univariate Cox regression analyses. For the multivariate Cox regression analysis, patient age and tumor size were excluded (*p* > 0.05). No adjustments were made for multiple comparisons.

### 2.2 FUS Regulates the Back Splicing of circTBC1D14 and Facilitates Hypoxia‐Induced SG Formation with circTBC1D14

**Figure 2:**
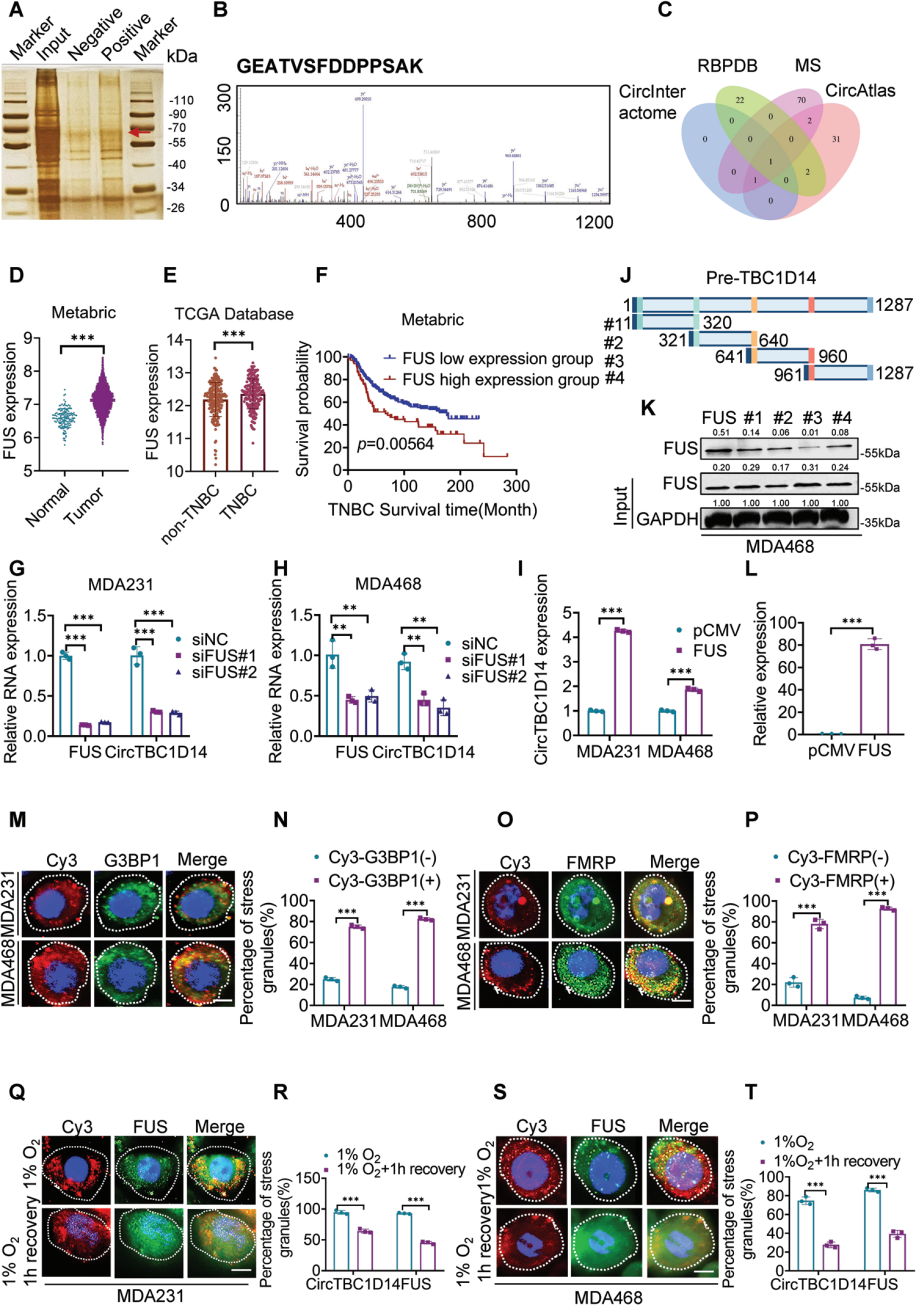
FUS regulated the back splicing of circTBC1D14 and contributed to hypoxia‐induced stress granules formation with circTBC1D14. A) Silver staining images of PAGE gels of circTBC1D14/proteins complex from pull‐down experiments of MDA231 cells. The red arrow represented the specific protein bands in the pull‐down complex by circTBC1D14 sense sequence compared with the antisense sequence. B) The peak map of FUS from the RNA pull‐down mass spectrometry. C) Venn diagram of the bioinformatic strategies from indicated databases and our RNA pulldown mass spectrometry. D) FUS expression analysis in the Metabric database. Two‐tailed unpaired t‐test. E) FUS expression analysis in TCGA database. Two‐tailed unpaired t‐test. F) Kaplan–Meier survival analysis of breast cancer patients from the Metabric database according to the expression level of FUS using the log‐rank test (*n* = 419). G and H) RT‐qPCR of circTBC1D14 and FUS expression with FUS silence in MDA231 and MDA468 cells. Two‐tailed unpaired t‐test. I) RT‐qPCR of circTBC1D14 expression with FUS overexpression in MDA231 and MDA468 cells. Two‐tailed unpaired t‐test. J) Schematic diagram of truncated pre‐TBC1D14 biotin‐labeled probes. K) Western blot of RNA pull‐down assays. L) RT‐qPCR analysis of RNA immunoprecipitation (RIP) assay in MDA231 cells. Two‐tailed unpaired t‐test. M,N) Immunofluorescence of endogenous circTBC1D14 and SGs (G3BP1) after 8 h hypoxic treatment in MDA231 and MDA468 cells. Right, quantification of the percentage of SGs (G3BP1+) co‐localizing with foci of circTBC1D14. Scale bar, 10 µm. Two‐tailed unpaired t‐test. O,P) Immunofluorescence of endogenous circTBC1D14 and SGs (FMRP) after 8 h hypoxic treatment in MDA231 and MDA468 cells. Right, quantification of the percentage of SGs (G3BP1+) co‐localizing with foci of circTBC1D14. Scale bar, 10 µm. Two‐tailed unpaired t‐test. Q,R) Immunofluorescence of endogenous circTBC1D14 and SGs (FUS) after 8 h hypoxic treatment or 1 h after removal of hypoxia. Right, the percentage of circTBC1D14 and FUS in MDA231cells containing SGs 8 h hypoxic treatment, or 1 h after removal of hypoxia. Scale bar, 10 µm. Two‐tailed unpaired t‐test. S,T) Immunofluorescence of endogenous circTBC1D14 and SGs (FUS) after 8 h hypoxic treatment or 1 h after removal of hypoxia. Right, the percentage of circTBC1D14 and FUS in MDA231 cells containing SGs 8 h hypoxic treatment, or 1 h after removal of hypoxia. Scale bar, 10 µm. Two‐tailed unpaired t‐test. The data are shown as the mean ± SD, NS (no significance) *p < 0.05 **p < 0.01, ***p < 0.001.

To further explore the regulation pattern and molecular mechanism of circTBC1D14, RNA pull‐down and mass spectrometry assays were performed to identify the potential binding proteins of circTBC1D14 using biotin‐labeled circTBC1D14 sense and antisense probes (**Figure** **2**A). Venn diagrams of three RBP databases revealed FUS as a crucial candidate RBP for circTBC1D14, and our mass spectrometry results also revealed FUS as a potential interacting protein with circTBC1D14 in MDA231 cells (Figure 2B,C). Analysis of the data from Metabric and The Cancer Genome Atlas (TCGA) databases revealed that FUS expression levels were significantly higher in breast cancer tissues than in normal tissues, suggesting its potential as an oncogene in breast cancer (Figure 2D; Figure S1C, Supporting Information). TCGA and GEO datasets showed that the expression levels of FUS were upregulated in TNBC tissues compared to those in the adjacent normal tissues (Figure 2E; Figure S1D, Supporting Information). These results demonstrate that FUS plays an essential role in breast cancer, especially TNBC. Additionally, the Metabric database analysis revealed that patients with TNBC exhibiting high FUS expression showed poor overall survival (Figure 2F). FUS is a key regulator that promotes the back splicing of circRNAs.^[^ ^11^ ^]^ Here, we performed RT‐qPCR and found a positive relationship between circTBC1D14 and FUS levels in MDA231 and MDA468 cells with ectopically overexpressed or knockdown FUS in MDA231 and MDA468 cells (Figure 2G–I; Figure S1E, Supporting Information). According to the prediction of the CircInteractome database (https://circinteractome.nia.nih.gov/), the FUS‐binding region was found in the 3′‐flanking intron of circTBC1D14 (Figure S1F, Supporting Information). To explore the key regulators of FUS, we designed a specific biotin‐labeled probe containing 1287 nucleotides and performed an RNA pull‐down assay. The results showed that the pre‐TBC1D14 probe captured the FUS protein more effectively than the antisense probe (Figure S1G, Supporting Information). To further investigate the binding region of FUS, we designed four truncated biotin‐labeled probes using the pre‐TBC1D14 probe. RNA pulldown assays were performed, and the results indicated that the first, second, and fourth truncated probes of pre‐TBC1D14 were significantly enriched by FUS in MDA468 cells (Figure 2J,K). An RNA immunoprecipitation (RIP) assay was also performed to confirm the interaction between FUS and pre‐CircTBC1D14 (Figure 2L). All results confirmed that FUS could bind to the 3′‐flanking intron region of pre‐TBC1D14 to promote the back splicing of circTBC1D14.

TNBC is more likely to have a hypoxic tumor microenvironment; as hypoxia is a key factor that contributes to the mechanism of oncogenesis and metastasis in TNBC.^[^ ^12^ ^]^ SGs are cytoplasmic membrane‐less ribonucleoprotein condensates with non‐membrane‐bound cellular compartments, which contribute to various environmental stresses, such as oxidative stress, heat shock, osmotic stress, and nutrient starvation.^[^ ^13^ ^]^ Proteomic analysis has revealed that 50% of SG components are a subset of RBPs, including FUS, G3BP1, TDP‐43, and FMRP.^[^ ^14^ ^]^ Many studies have suggested that FUS plays a key role in SG formation.^[^ ^15^ ^]^ We investigated whether the mimetic hypoxic microenvironment of TNBC could induce SG formation in FUS–circTBC1D14. Interestingly, we discovered that circTBC1D14 and SG‐associated proteins, G3BP1 and FMRP, could co‐localize in the cytoplasm to form SGs upon exposure to oxidative stress induced by 1% O_2_ in MDA231 and MDA468 cells (Figure 2M,O), and circTBC1D14 was recruited to 82.3 and 92.6% of G3BP1+ SGs and FMRP+ SGs in MDA468 cells, respectively (Figure 2N,P). These results were consistent with the putative interactions of the FUS–circTBC1D14 complex with SGs in MDA231 and MDA468 cells (Figure 2Q–T; Figure S1I,J, Supporting Information). These findings revealed that the hypoxia‐induced FUS–circTBC1D14 complex could play a role in SGs and that the localization of FUS and circTBC1D14 changed under hypoxic conditions, which requires further exploration.

Based on these results, we explored the potential roles of circTBC1D14 in MDA231 and MDA468 cells pre‐treated under hypoxia. We designed small interfering RNAs (siRNAs) and a circTBC1D14 overexpression plasmid and verified the transfection efficiency in MDA231 and MDA468 cells using RT‐qPCR (Figure S1K,L, Supporting Information). To explore tumor progression in TNBC cells, we performed 3‐(4,5‐dimethylthiazol‐2‐yl)‐2,5‐diphenyl tetrazolium bromide (MTT) assays and found that the overexpression of circTBC1D14 could promote cell proliferation, while its silencing inhibited the cell viability (Figure S2A,B, Supporting Information). We also found that overexpression of circTBC1D14 significantly facilitated clone formation (Figure S2C,D, Supporting Information). Next, we performed a 5‐ethynyl‐2′‐deoxyuridine (EdU) assay and found that the proliferation process was increased by the upregulation of circTBC1D14 expression and significantly inhibited by its silencing (Figure S2E,F, Supporting Information). In addition, transwell and wound healing assays indicated that circTBC1D14 could promote the metastasis of MDA231 and MDA468 cells, while its downregulation could suppress this effect (Figure S2G–J, Supporting Information). Our results show that circTBC1D14 acts as an oncogene and facilitates tumor progression in TNBC cells under hypoxic conditions.

### 2.3 Hypoxia‐Induced PRMT1 Facilitates the Subcellular Localization and Formation of FUS–circTBC1D14‐Associated SGs

**Figure 3:**
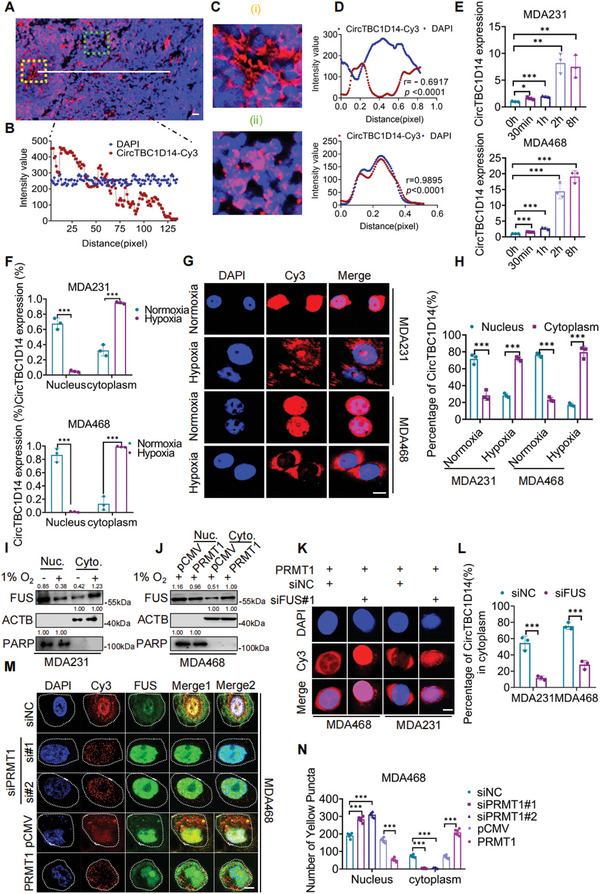
Hypoxia‐induced PRMT1 contributed to subcellular localization and formation of FUS‐circTBC1D14‐associated stress granules. A,B) FISH of xenografts in nude mice with circTBC1D14. Scale bars = 50 µm. Right, the intensity variability is calculated with ImageJ. The red region showed the distribution of the circTBC1D14 probe, and the blue region showed the nuclei staining by DAPI. C,D) circTBC1D14 staining in different areas of sections from (A). The yellow region indicates the center of the tumor simulating the hypoxic area, while the green region indicates the margin of the tumor simulating the normoxic area. Right, the intensity variability of the yellow region and green region is calculated with ImageJ. E) RT‐qPCR analysis of circTBC1D14 with hypoxia in time gradient in MDA231 and MDA468 cells. Two‐tailed unpaired t‐test. F) Relative distribution of circTBC1D14 in MDA231 and MDA468 cells with hypoxia treatment or not determined by RT‐qPCR. U6 served as the nuclear RNA marker and GAPDH served as the cytoplasmic RNA marker. Two‐tailed unpaired t‐test. G,H) RNA FISH assays of circTBC1D14 in MDA231 and MDA468 cells. The red region showed the distribution of the circTBC1D14 probe, and the blue region showed the nuclei staining by DAPI. Scale bar, 10 µm. Right, statistic diagram. Two‐tailed unpaired t‐test. I) Western blot of FUS relative distribution in normoxic or hypoxic conditions. J) Western blot of FUS relative distribution with PRMT1 overexpression or not. K,L) FISH of circTBC1D14 in PRMT‐stable expression of MDA231 and MDA468 cells with FUS knockdown or not in hypoxic conditions. Right, statistic diagram. Scale bar, 10 µm. Two‐tailed unpaired t‐test. M,N) Immunofluorescence of co‐localized FUS‐circTBC1D14 SGs in MDA468 cells with PRMT1 overexpression or knockdown in hypoxic conditions. Right, statistic diagram. Scale bar, 10 µm. Two‐tailed unpaired t‐test. The data are shown as the mean ± SD, NS (no significance) *p < 0.05 **p < 0.01, ***p < 0.001.

To explore and verify the relationship between circTBC1D14 and hypoxic conditions, we found that the circTBC1D14 probe was highly expressed in the central areas of xenografts, simulating a hypoxic environment (**Figure** **3**A,B). Furthermore, we observed that circTBC1D14 in hypoxic central areas in xenografts was mostly localized in the cytoplasm. In contrast, we observed that circTBC1D14 nearly coincided with the nucleus at tumor margins away from the hypoxic central regions (Figure 3C,D). This result indicated a positive relationship between circTBC1D14 and hypoxia. We found that circTBC1D14 was highly expressed in TNBC; therefore, we assumed that hypoxia might play a role in regulating circTBC1D14. We also found that circTBC1D14 was significantly increased by hypoxic treatment (Figure 3E). Furthermore, RT‐qPCR and FISH assays indicated that hypoxia‐induced circTBC1D14 was more localized in the cytoplasm (Figure 3F–H). Gene Ontology analysis from our mass spectrometry also revealed that circTBC1D14‐associated proteins were crucial for RNA nucleocytoplasmic transport and localization, in which FUS played an essential role (Figure S1H, Supporting Information). This indicates that FUS may contribute to the subcellular localization of circTBC1D14 in TNBC. Interestingly, compared to normoxic conditions, hypoxia‐induced FUS protein was more localized in the cytoplasm, which was consistent with the translocation of circTBC1D14 (Figure 3I). It has been reported that the localization of FUS protein can be regulated by arginine methylation and that PRMT1 protein is the key factor in the export of FUS protein to the cytoplasm.^[^ ^16^ ^]^ Our results showed that the FUS protein was more likely to localize in the cytoplasm with PRMT1 overexpression under hypoxic conditions (Figure 3J). In addition, we knock down FUS in MDA231 and MDA468 cells with PRMT1 overexpression and found circTBC1D14 could not be transferred to the cytoplasm under hypoxic conditions (Figure 3K,L). These results demonstrated that circTBC1D14 expression was upregulated under hypoxic conditions and that PRMT1 contributed to the translocalization of the FUS–circTBC1D14 complex. We then investigated whether PRMT1 regulates the SGs of FUS–circTBC1D14 condensates under hypoxic conditions. We knock down PRMT1 with siRNA and found that FUS–circTBC1D14‐associated SGs were decreased under hypoxic conditions, while upregulated PRMT1 expression enhanced SG formation by FUS–circTBC1D14 in TNBC cells. (Figure 3M,N). Our results indicated that PRMT1 positively regulates the SGs of FUS–circTBC1D14 under hypoxic conditions. In conclusion, hypoxia‐induced PRMT1 expression plays an essential role in the formation of FUS–circTBC1D14 SGs.

### 2.4 circTBC1D14 Facilitates TNBC Progression by Inhibiting the Ubiquitination and Degradation of PRMT1 Under Hypoxic Conditions

**Figure 4:**
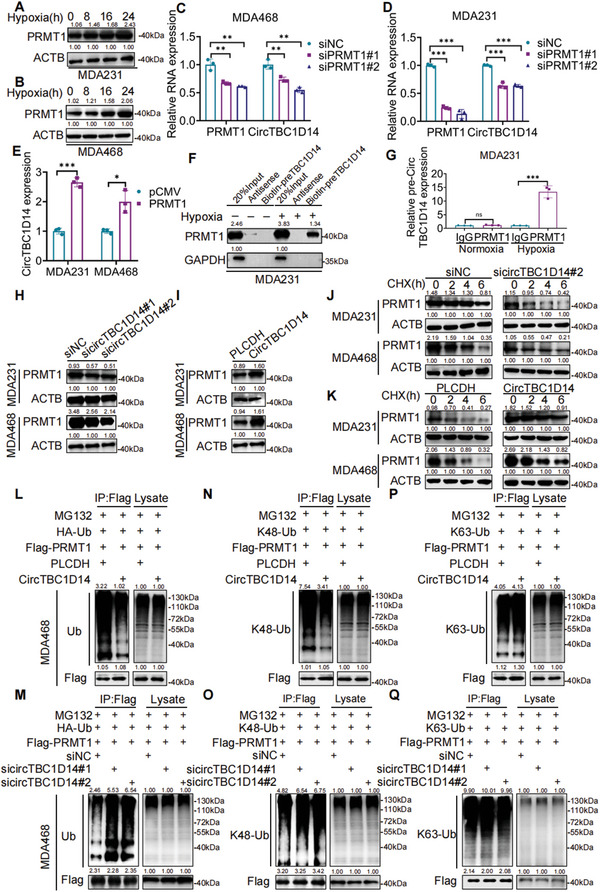
CircTBC1D14 facilitated TNBC progression by inhibiting the ubiquitination and degradation of PRMT1 in hypoxic conditions. A,B) Western blot of PRMT1 with hypoxia treatments. Right, protein gray values of PRMT1 and ACTB were calculated with ImageJ. C–E) Quantitative real‐time PCR analysis of RNA expression in MDA468 or MDA231 cells with PRMT1 overexpression or knockdown. Statistical significance was determined by a two‐tailed unpaired t‐test. F) Western blot of RNA pull‐down assays. G) RT‐qPCR analysis of RNA immunoprecipitation (RIP) assay in MDA231 cells. Two‐tailed unpaired t‐test. H,I) Western blot analysis of circTBC1D14 overexpression or knockdown in MDA231 and MDA468 cells. J,K) Western blot analysis of the half‐life of PRMT1 in MDA231 and MDA468 cells transfected with circTBC1D14 overexpression or knockdown treated with the protein synthesis inhibitor CHX (400 µg mL^−1^). L–Q) Ubiquitination of exogenous PRMT1 in MDA468 cells transfected with indicated plasmids based on circTBC1D14 overexpression (up) or circTBC1D14 knockdown (below) with MG132 (20 um) pretreatment. Western blot was detected with indicated antibodies. The data are shown as the mean ± SD, NS (no significance) *p < 0.05 **p < 0.01, ***p < 0.001.

We found that circTBC1D14 expression was upregulated over time under hypoxia (Figure 3E). Interestingly, PRMT1 protein levels were also increased under hypoxic conditions in MDA231 and MDA468 cells (**Figure** **4**A,B). To explore the relationship between circTBC1D14 and PRMT1, we performed an RT‐qPCR assay and found that the levels of PRMT1 mRNA remained unchanged in the TNBC cell line with either circTBC1D14 overexpression or knockdown (Figure S3A,B, Supporting Information). However, we found that PRMT1 could upregulate circTBC1D14 expression, while its silencing significantly decreased circTBC1D14 levels in MDA231 and MDA468 cells after hypoxia pretreatment (Figure 4C–E). RNA pull‐down and RIP assays also showed that circTBC1D14 could significantly interact with PRMT1 only in hypoxia, but not normoxia in MDA231 cells (Figure 4F,G). Compared to our previous results, we considered that PRMT1 might replenish the function of FUS to promote the back‐splicing of circTBC1D14 under hypoxic conditions. In addition, we found that overexpression of ectopic circTBC1D14 increased PRMT1 protein expression, whereas silenced circTBC1D14 decreased PRMT1 protein expression (Figure 4H,I). Accumulating evidence has revealed that circRNAs play a crucial role in regulating protein stability.^[^ ^17^ ^]^ After treatment with the protein synthesis inhibitor cycloheximide (CHX), circTBC1D14 overexpression increased the half‐life of the PRMT1 protein, but circTBC1D14 silencing accelerated the degradation of PRMT1 in MDA231 and MDA468 cells (Figure 4J,K). There are two major proteolytic systems in eukaryotic cells, ubiquitination, and autophagy. Substrates conjugated with ubiquitin induce degradation by the proteasome.^[^ ^18^ ^]^ Autophagy regulates the cleaning of cytoplasmic constituents by the lysosome.^[^ ^19^ ^]^ Thereafter, we treated circTBC1D14‐downregulated and‐controlled MDA468 cells with MG132 based on CHX treatment in a time gradient, and the results indicated that circTBC1D14 could regulate the stability of PRMT via the ubiquitination pathway (Figure S3D, Supporting Information). Thus, we explored whether circTBC1D14 was involved in modulating PRMT1 ubiquitination. We transfected ectopic HA‐Ub, Flag‐PRMT1, PLCDH, and circTBC1D14, as indicated by proteasome inhibitor MG132 treatment, and the ubiquitination of exogenous PRMT1 was significantly reduced in circTBC1D14‐overexpressed cells (Figure 4L). Consistently, we knock down circTBC1D14 markedly increased the accumulation of ubiquitinated Flag‐PRMT1 in MDA468 cells (Figure 4M). Thus, we next explored circTBC1D14 effects on the ubiquitination of PRMT1 by K48 or K63 polyubiquitination. Afterward, we transfected ectopic K48‐Ub, Flag‐PRMT1, PLCDH, and circTBC1D14, as indicated by proteasome inhibitor MG132 treatment, and found that K48‐ubiquitination of exogenous PRMT1 was significantly reduced in circTBC1D14‐overexpressed cells (Figure 4N). Consistently, we knock down circTBC1D14 markedly increased the accumulation of K48‐ubiquitinated Flag‐PRMT1 in MDA468 cells (Figure 4O). In contrast, we also transfected ectopic K63‐ub, Flag‐PRMT1, PLCDH, and circTBC1D14, as indicated by proteasome inhibitor MG132 treatment, and the K63‐ubiquitination of exogenous PRMT1 in circTBC1D14‐overexpressed cells was nearly similar to that in the control group (Figure 4P). Knockdown circTBC1D14 lost the potential to regulate K63‐ubiquitinated Flag‐PRMT1 in MDA231 and MDA468 cells, and K63‐associated polyubiquitination levels were not significantly different among the indicated groups (Figure 4Q). Overall, our results confirmed that circTBC1D14 could stabilize PRMT1 protein by inhibiting K48‐associated polyubiquitination.

Studies have demonstrated that PRMT1 expression is upregulated in TNBC and contributes to tumor progression and chemotherapy resistance.^[^ ^20^ ^]^ Metabric and TCGA databases also indicated that PRMT1 expression is upregulated in breast cancer, especially in TNBC (Figure S4A–C, Supporting Information). We then performed clone formation and MTT assays in TNBC cells before hypoxic treatment and found that the overexpression of PRMT1 could diminish the inhibition of cancer cell proliferation by downregulating circTBC1D14 expression (Figure S4D,E, Supporting Information). In addition, the transwell assay revealed that PRMT1 could reverse the suppression of cancer cell metastasis caused by circTBC1D14 silencing (Figure S4F,G, Supporting Information). In summary, circTBC1D14 plays a significant role in TNBC progression and development by regulating PRMT1 expression.

### 2.5 circTBC1D14 Stabilizes Hypoxia‐Inducible Factor‐1‐Alpha (HIF‐1a) Expression by Inhibiting the Translation of von Hippel–Lindau (VHL)

Considering FUS–circTBC1D14 SG formation, we assumed that circTBC1D14 might contribute to its tumor progression function through the granules under hypoxic conditions, among which HIF‐1*α* is an essential element. Once HIF‐1*α* is hydroxylated, it is recognized and marked for proteasome degradation by von Hippel–Lindau (VHL) tumor suppressor protein, an E3 ubiquitin ligase.^[^ ^21^ ^]^ To explore the role of circTBC1D14 in the axis of HIF‐1*α*‐VHL, we performed western blot and found that upregulated circTBC1D14 could increase HIF‐1*α* protein and decrease VHL protein in the nucleus and cytoplasm, and downregulated circTBC1D14 decreased HIF‐1*α* protein and increase VHL protein (Figure S5A,B, Supporting Information), whereas VHL mRNA expression levels did not significantly change with circTBC1D14 upregulation or downregulation (Figure S5C,D, Supporting Information). These results suggest that the differential expression of the VHL protein was not due to the influence of circTBC1D14 on VHL gene expression. Moreover, RNA FISH of VHL and circTBC1D14 combined with immunofluorescence of G3BP1 in hypoxia‐treated TNBC cells showed that VHL RNA, circTBC1D14 probe, and G3BP1 co‐localized in SGs (Figure S5E,F, Supporting Information). In addition, immunofluorescence analysis of VHL and G3BP1/circTBC1D14 revealed that the VHL protein was not co‐localized in SGs (Figure S5G,H, Supporting Information). Therefore, we speculated that circTBC1D14 may affect the expression level of the VHL protein by regulating VHL translation. Furthermore, Western blot analysis showed the expression of VHL protein was upregulated with circTBC1D14 silencing and downregulated with circTBC1D14 overexpression in whole cell lysis from MDA231 and MDA468 cells, Western blot analysis revealed that the translation of VHL was inhibited in hypoxia‐treated cells, and our results showed that silence of circTBC1D14 could decrease eIF2*α* expression and overexpressed circTBC1D14 could increase eIF2*α* expression, which functions as an inhibitor for translation initiation of VHL.^[^ ^22^ ^]^ We also found circTBC1D14 contributed to regulating Ran protein, which plays an important role in the nucleus import of HIF‐1*α*.^[^ ^23^ ^]^ These findings indicate that the translation of VHL can be inhibited by circTBC1D14 under stress and circTBC1D14 might contribute to subcellar localization of HIF‐1*α* (Figure S5I,J, Supporting Information).

### 2.6 FUS–circTBC1D14 SGs Facilitate Lysosome‐Associated Autophagy Flux

**Figure 5:**
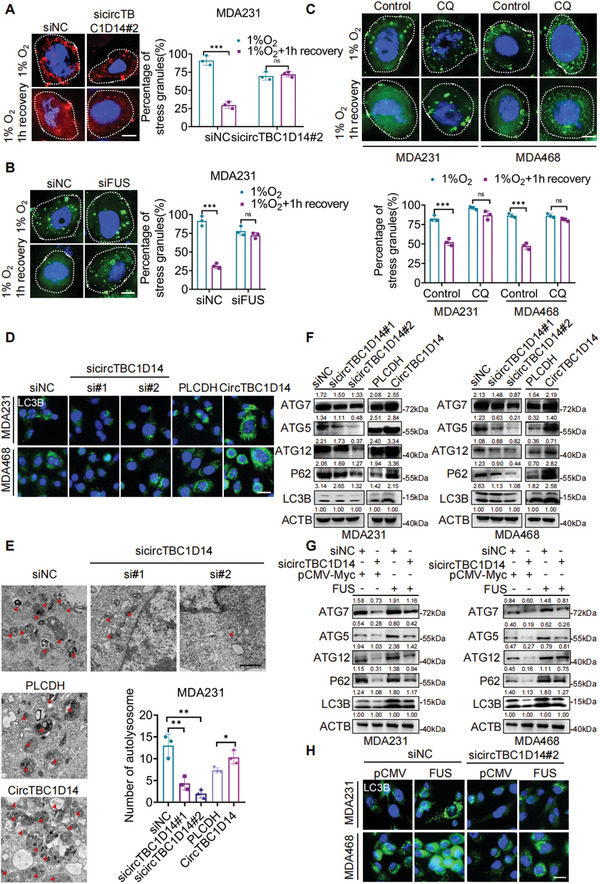
FUS‐circTBC1D14 stress granules contributed to lysosome‐associated autophagy flux. A) Immunofluorescence of SGs (G3BP1) in MDA231 cells with control or sicircTBC1D14 after 8 h hypoxic treatment or 1 h after removal of hypoxia. Right, percentage of cells containing SGs in cells. Scale bar, 10 µm. Two‐tailed unpaired t‐test. B) Immunofluorescence of SGs (G3BP1) in MDA231 cells with control or siFUS after 8 h hypoxic treatment or 1 h after removal of hypoxia. Right, percentage of cells containing SGs in cells. Scale bar, 10 µm. Two‐tailed unpaired t‐test. C) Immunofluorescence of SGs (G3BP1) in MDA231 cells with control or chloroquine (CQ) after 8 h hypoxic treatment, or 1 h after removal of hypoxia. Below, is the percentage of cells containing SGs in cells. Scale bar, 10 µm. Two‐tailed unpaired t‐test. D) Immunofluorescence of LC3B in MDA231 and MDA468 cells with circTBC1D14 overexpression or knockdown. Scale bar, 20 µm. E) Electron microscopy images of MDA231 cells with circTBC1D14 overexpression or knockdown. Scale bar,500 nm. Statistical diagram of autolysosomes in E). Two‐tailed unpaired t‐test. F) Western blot of MDA231 and MDA468 cells with circTBC1D14 overexpression or knockdown. G) Western blot of MDA231 and MDA468 cells with circTBC1D14 knockdown while with FUS overexpression. H) Immunofluorescence of LC3B in MDA231 and MDA468 cells with circTBC1D14 knockdown while with FUS overexpression. Scale bar, 20 µm. The data are shown as the mean ± SD, NS (no significance) *p < 0.05 **p < 0.01, ***p < 0.001.

Our results have demonstrated the formation of FUS–circTBC1D14 SGs in hypoxic conditions (Figure 2M–T). Previous studies have indicated that SGs are dynamic structures with rapid exchange rates of components, which can be cleared by autophagy.^[^ ^24^ ^]^ Interestingly, we found that circTBC1D14 and FUS controlled the elimination of SGs. The results indicated that MDA231 cells depleted of circTBC1D14 or FUS formed similar numbers of SGs induced by hypoxia compared to control cells. In contrast, during recovery from stress (1 h recovery after hypoxic treatment), cells treated with siRNA targeting circTBC1D14 or FUS failed to eliminate SGs (**Figure** **5**A,B), which closely mimicked the function of autophagy inhibition with chloroquine (CQ) (Figure 5C). These results suggest that circTBC1D14 and FUS might contribute to triggering SGs to enter autophagy to maintain cell homeostasis under hypoxic conditions. The immunofluorescence image showed that upregulated circTBC1D14 expression increased light chain 3B (LC3B) expression, whereas its silencing decreased LC3B expression in MDA231 and MDA468 cells (Figure 5D). Electron microscopy also revealed that upregulated circTBC1D14 increased cellular autolysosome and downregulated circTBC1D14 decreased autolysosome formation in MDA231 cells (Figure 5E). Western blotting showed that circTBC1D14 could regulate the expression levels of autophagy‐related Atg‐5, Atg7, Atg12, P62, and LC3B, which contribute to autophagic vesicles associated with membrane elongation (Figure 5F). These results revealed that circTBC1D14 plays an essential role in lysosome‐associated autophagy. Western blot and immunofluorescence also found that FUS could participate in circTBC1D14‐regulated autophagy and rescue the autophagy suppression effect caused by circTBC1D14 downregulation (Figure 5G,H). Overall, these findings confirmed that FUS and circTBC1D14 form a complex that can dock on SGs to control their elimination via autophagy under hypoxic conditions.

Many studies found that autophagy could contribute to either a pro‐survival or a pro‐death role under stress conditions.^[^ ^25^ ^]^ However, the possibility of autophagic cell death could not be excluded. Based mostly on our results that autophagy exerts cytoprotective effects in TNBC cells, it is essential to explore that circTBC1D14‐FUS ‐regulated cell death is cell death with autophagy or cell death by autophagy.^[^ ^26^ ^]^ To verify the effects of autophagy in circTBC1D14‐FUS‐related stress granules metabolism, we use the pharmacological agent most commonly used to suppress autophagy‐3‐methyladenine (3‐MA), which interferes with the formation of autophagosomes.^[^ ^25b^ ^]^ As shown in Figure S6A–D (Supporting Information), the knockdown circTBC1D14 effectively decreased reactive oxygen species (ROS) and mitochondrial membrane potential (Δ*ψ*m). Furthermore, we found that overexpression of circTBC1D14 led to a significant rescue of Δ*ψ*m and ROS under hypoxic conditions. In addition, we also found that 3‐MA treatment decreased Δ*ψ*m and ROS in TNBC cells under hypoxic conditions (Figure S6A–D, Supporting Information). Then we performed a cytotoxicity assay to detect cell death underlying 3‐MA treatment or not in hypoxic condition, and we found that downregulated circTBC1D14 increased cell death and upregulated circTBC1D14 decreased cell death in TNBC cells. Meanwhile, 3‐MA accelerated the cell death process and hampered the cytoprotective function of circTBC1D14 in TNBC cells under hypoxic conditions (Figures S3E–G and S6E,F, Supporting Information). In summary, circTBC1D14‐FUS stress granules‐related autophagy is considered to be a pro‐survival pathway in the dying cell, which is called cell death with autophagy.^[^ ^26^ ^]^ This is a way that the inhibition of autophagy does not alter the fate of the cell, but inhibition of autophagy could accelerate cell death. Therefore, circTBC1D14‐FUS regulated‐stress granules play a pro‐survival role through autophagy in TNBC cells.

Next, we examined whether circTBC1D14‐mediated autophagy could regulate TNBC cell proliferation and metastasis. We overexpressed circTBC1D14 in TNBC cells and then treated cells with 3‐MA to inhibit autophagy. And then we performed MTT, Edu, clone formation, and transwell assays and found that 3‐MA treatment inhibited circTBC1D14‐promoted proliferation and metastasis in TNBC cells under hypoxic conditions (Figure S7A–H, Supporting Information). implying that autophagy plays an essential role in TNBC growth and metastasis in circTBC1D14‐FUS‐related stress granules system. We then performed a western blot assay and the results indicated that 3‐MA could inhibit the autophagy‐related protein level of Atg5, Atg7, Atg12, P62, and LC3B regulated by circTBC1D14. Furthermore, under consideration of the possibility of autophagic cell death, we also use autophagy inhibitor chloroquine (CQ), which inhibited the fusion of lysosomes with autophagosomes, and our results showed that circTBC1D14 could significantly regulate autophagy in TNBC cells with hypoxia treatments. All these results demonstrated that circTBC1D14 regulates TNBC growth and metastasis via autophagy (Figure S7I,J, Supporting Information).

### 2.7 circTBC1D14 Facilitates Lysosome‐Associated Autophagy Flux as a Scaffold to Reinforce the Interaction Between FUS and LAMP1

**Figure 6:**
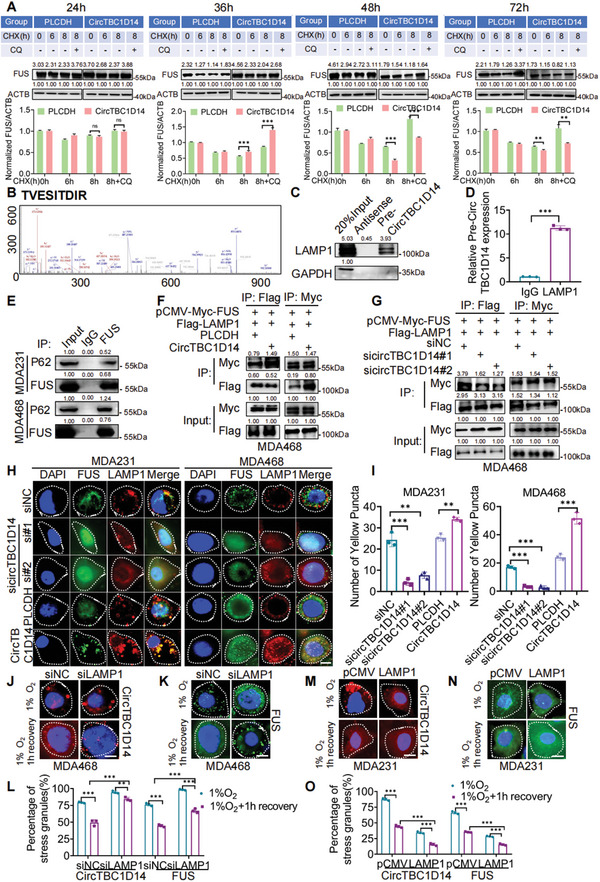
CircTBC1D14 facilitated lysosome‐associated autophagy flux as a scaffold to reinforce the interaction between FUS and LAMP1. A) Western blot of MDA468 cells with circTBC1D14 and PLCDH expression following treatment with CHX or MG132. Below, are protein gray values of FUS normalized by ACTB. B) The peak map of LAMP1 from the RNA pull‐down mass spectrometry. C) Western blot of RNA pull‐down assays pretreated with hypoxia. D) RT‐qPCR analysis of RNA immunoprecipitation (RIP) assay in MDA468 cells pretreated with hypoxia. Two‐tailed unpaired t‐test. E) Co‐IP assay of FUS and P62 in MDA231 and MDA468 cells in hypoxic conditions. F,G) Co‐IP assay of LAMP1 and FUS in MDA468 cells with circTBC1D14 overexpression or circTBC1D14 knockdown pretreated with hypoxia. H) Immunofluorescence of co‐localized FUS and LAMP1 with circTBC1D14 overexpression or knockdown in MDA231 and MDA468 cells pretreated with hypoxia. Scale bar, 10 µm. I) Statistical diagram of yellow puncta in (H). J–L) Immunofluorescence of SGs (circTBC1D14 or FUS) in MDA468 cells with control or siLAMP1 after 24 h hypoxic treatment or 1 h after removal of hypoxia. Scale bar, 20 µm. Percentage of cells containing SGs (circTBC1D14 or FUS) in cells. Two‐tailed unpaired t‐test. M–O) Immunofluorescence of SGs (circTBC1D14 or FUS) in MDA468 cells with control or LAMP1 overexpression after 24 h hypoxic treatment or 1 h after removal of hypoxia. Scale bar, 20 µm. Percentage of cells containing SGs (circTBC1D14 or FUS) in cells. Two‐tailed unpaired t‐test. The data are shown as the mean ± SD, NS (no significance) *p < 0.05 **p < 0.01, ***p < 0.001.

Our findings demonstrate that hypoxia‐induced FUS–circTBC1D14 SGs contribute to lysosome‐associated autophagy flux. In **Figure** **6**A, our findings demonstrate that hypoxia‐induced FUS–circTBC1D14 SGs contribute to lysosome‐associated autophagy flux. The CHX chase experiment showed that basal lysosome‐dependent degradation of FUS was reduced. Based on CHX chase experiments, we also treated cells with CQ, a fusion inhibitor of autophagosomes and lysosomes, and we found there is no difference in FUS expression between PLCDH and circTBC1D14 group, which indicated circTBC1D14 regulated autophagy did not initiate in the lysosome‐dependent clearance of FUS in 24 h; and then, in particular, circTBC1D14 exerted the enhanced effect of lysosome‐dependent clearance of FUS, and upregulated circTBC1D14 promoted the clearance on FUS after treatment with hypoxia between 36–48 h; finally in 72 h, circTBC1D14‐regulated autophagy has finished the clearance on FUS. These results indicated that hypoxia‐induced circTBC1D14 plays a role in the lysosome‐dependent clearance of FUS and there are multiple and dynamic processes of circTBC1D14‐regulated autophagy on FUS (Figure 6A). Subsequently, we hypothesized that circTBC1D14 regulates autophagy by recruiting lysosomes. Our mass spectrometry results indicated that LAMP1 might interact with circTBC1D14 in the cytoplasm (Figure 6B). Both RNA pull‐down and RIP assays confirmed that circTBC1D14 interacted with LAMP1(Figure 6C,D). Next, we explored the relationship among LAMP1–FUS–circTBC1D14. Previous evidence has confirmed that FUS could interact with P62,^[^ ^27^ ^]^ and our results also demonstrated that there is an interaction between FUS and P62 in TNBC cells with hypoxia treatments (Figure 6E). We then performed a western blot assay and found knockdown circTBC1D14 could decrease LAMP1 in TNBC cells (Figure S7K, Supporting Information). Co‐IP assay and immunofluorescence staining verified that circTBC1D14 enhanced the interaction between FUS and LAMP1 under hypoxic conditions (Figure 6F–I). We also performed immunofluorescence in MDA468 cells with downregulated LAMP1, and results showed that circTBC1D14‐positive SGs and FUS‐positive SGs induced by hypoxia could not be cleaned timely, which might lead to inhibition of autophagy and result in cell damage (Figure 6J–L). In contrast, during recovery from stress (1 h recovery after hypoxic treatment), knockdown LAMP1 targeting circTBC1D14 or FUS failed to eliminate the SGs compared with the siNC group (Figure 6J–L). Meanwhile, immunofluorescence in MDA231 cells showed that circTBC1D14‐positive SGs and FUS‐positive SGs induced by hypoxia was decreased with the LAMP1 overexpression, which might enhance the autophagy process (Figure 6J–L). In contrast, during recovery from stress (1 h recovery after hypoxic treatment), upregulated LAMP1 targeting circTBC1D14 or FUS could diminish the SGs compared with the pCMV group (Figure 6M–O). These results revealed that LAMP1 could control the elimination of FUS–circTBC1D14‐associated SGs. Therefore, circTBC1D14 contributes to lysosome‐associated autophagy flux by recruiting LAMP1 and enhancing the clearance of SGs to maintain cellular homeostasis under hypoxic conditions.

### 2.8 circTBC1D14 Promotes Tumor Growth and Lung Metastasis In Vivo

**Figure 7:**
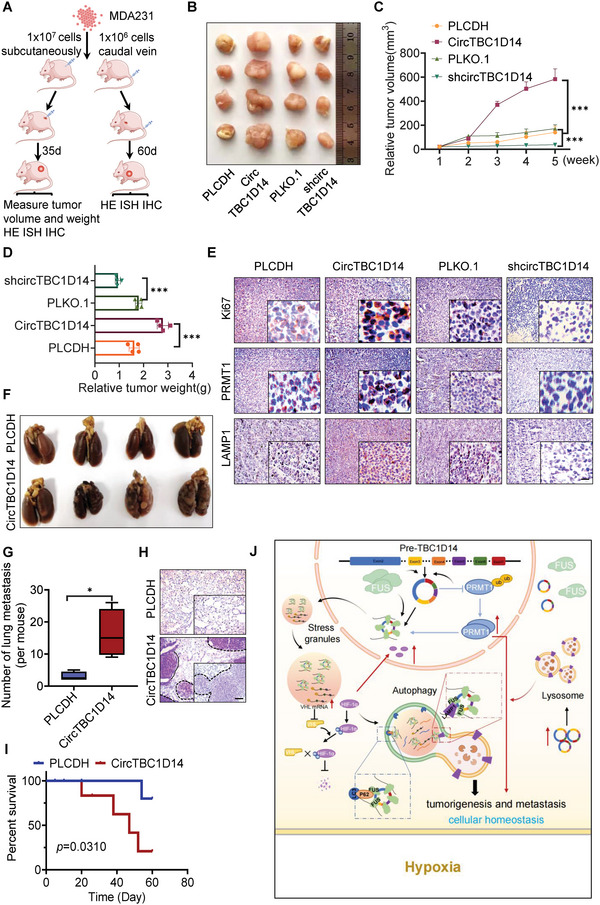
CircTBC1D14 promoted tumor growth and lung metastasis in vivo. A) Schematic diagram of xenografts in BALB/c nude mice by inoculating MDA231 cells co‐transfected with stable expression of PLCDH, circTBC1D14, sh‐NC, and sh‐circTBC1D14, respectively, at their flanks or tail vein injection. B) Representative images of xenograft tumors. (*n* = 4, each group). C) Mean tumor volumes on days gradient for each group of xenografts in nude mice. (*n* = 4, mean ± s.d). D) Tumor weight of each group of xenografts in nude mice. (*n* = 4, mean ± s.d) E) Immunohistochemistry (IHC) assay of protein expression of Ki‐67, PRMT1, and LAMP1 in tumors from each group of xenografts in nude mice. Scale bars = 100 µm. F) Representative images of pulmonary surface nodules. G) Statistical diagram of (F). H) Hematoxylin and eosin (H&E) of staining in pulmonary surface nodules of the indicated circTBC1D14 genotypes. Scale bars = 100 µm. I) Animal survival of circTBC1D14 overexpression and control group. (*n* = 8, each group). Log‐rank test. J) Schematic illustration of the roles and molecular mechanisms of circTBC1D14 in hypoxic conditions. CircTB1D14 could interact with FUS and PRMT1, which could induce circTBC1D14 cyclization and expression with hypoxia treatments. Then FUS‐circTBC1D14‐associated SGs were transferred to the cytoplasm after being modified by hypoxia‐induced PRMT1. In turn, circTBC1D14 increased the stability of PRMT1 by inhibiting its K48‐regulated polyubiquitination, leading to enhanced nucleus transport. Meanwhile, VHL mRNA could co‐localize in FUS‐circTBC1D14 positive‐stress granules, leading to an inhibition of VHL translation, which enhanced HIF‐1*α* overexpression and import to the nucleus. In addition, FUS‐circTBC1D14 could initiate a cascade of SGs‐linked proteins to recognize and control the elimination of stress granules (SGs) by recruiting LAMP1 and enhancing the lysosome‐associated autophagy flux in TNBC. The data are shown as the mean ± SD, NS (no significance) *p < 0.05 **p < 0.01, ***p < 0.001.

To investigate the role of circTBC1D14 in vivo, we implanted MDA231 cells with stable overexpression or knockdown circTBC1D14 and control cells into the flanks of BALB/c mice. Knockdown circTBC1D14 significantly decreased the tumorigenicity and vice versa (**Figure** **7**A,B). In addition, overexpression of circTBC1D14 resulted in a remarkable increase in tumor volume and weight. Consistently, the knockdown circTBC1D14 significantly decreased tumor growth (Figure 7C,D). Immunohistochemistry (IHC) staining of the harvested xenografts revealed that knockdown circTBC1D14 significantly decreased the expression of the proliferation biomarker Ki67, while its overexpression increased Ki67 expression (Figure 7E). PRMT1 and LAMP1 were also detected in the circTBC1D14 overexpression group by IHC staining (Figure 7E). We also explored the function of circTBC1D14 in breast cancer lung metastasis via the intravenous injection of MDA231 cells. Our results showed that circTBC1D14 overexpression aggravated lung colonization of breast cancer cells in nude mice (Figure 7F,G). Hematoxylin–eosin staining of the lungs revealed a large number of tumor nodes in the circTBC1D14 overexpression group (Figure 7H). Overexpression of circTBC1D14 with an increased number of lung metastatic nodes drastically shortened the lifespan of nude mice (Figure 7I). Together, these results confirmed that the circTBC1D14 could promote TNBC development and can be used as a potential diagnostic and therapeutic target in TNBC.

## 3 Discussion

CircRNA, which was first discovered 40 years ago, was initially thought to be formed by an RNA splicing error that lacked protein‐coding functions.^[^ ^28^ ^]^ Advancements in high‐throughput RNA sequencing and circRNA‐specific molecular tools revealed that circRNAs have many crucial biological functions in cancer progression. Specifically, circRNAs can serve as ceRNAs, sponging miRNAs to regulate the downstream gene activities,^[^ ^29^ ^]^ interacting with RBPs to affect gene expression,^[^ ^30^ ^]^ and encoding functional proteins that activate signaling pathways.^[^ ^31^ ^]^ Thus far, the characteristics of circRNAs interacting with RBPs have attracted extensive attention and have been most widely reported in breast cancer. For example, hsa_circ_0068631 can bind to the eukaryotic translation initiation factor 4A3 to maintain c‐Myc mRNA stability and positively regulate c‐Myc expression.^[^ ^30^ ^]^ Two RBPs (CASC3 and metatherian) can bind directly to the characterized motifs of circ‐NOL10, leading to circ‐NOL10 expression change.^[^ ^32^ ^]^ Activated transcriptionally by Krüppel‐like factor 5, RNA‐binding protein FUS can promote the back splicing of circROBO1, resulting in liver metastasis in breast cancer.^[^ ^9^ ^]^ Considering that the association between RBPs and circRNAs is a key orientation in the current study, it is necessary to explore and understand its molecular mechanism to provide a new direction and target for future treatment of breast cancer.

In our study, we identified and investigated a novel TNBC circRNA, circTBC1D14, which is derived from exon 2 ‐7 of the TBC1D14 gene. Upregulated circTBC1D14 was correlated with poor prognosis in patients with breast cancer, indicating that circTBC1D14 may serve as a potential prognostic biomarker for breast cancer. Meanwhile, circTBC1D14 was mostly upregulated in triple‐negative cell lines, such as MDA231 and MDA468 cells. Moreover, we confirmed that knockdown circTBC1D14 could significantly suppress tumor progressive phenotypes, such as cell proliferation, migration, and invasion in vitro, while downregulation of circTBC1D14 inhibited oncogenesis and metastasis in vivo, and vice versa.

To further explore the molecular mechanism of circTBC1D14 in TNBC, we took advantage of bioinformatics strategies of circRNA binding protein and RNA pull‐down‐based mass spectrometry, we only selected FUS as a potential binding protein from this intersection. As a classical RBP, many studies focus on FUS's function to be a crucial regulator of the biogenesis of circRNAs.^[^ ^33^ ^]^ For example, studies have demonstrated that FUS can bind to ZNF609 pre‐mRNA to increase circZNF609 expression in lung cancer.^[^ ^34^ ^]^ This study focused on the subcellular localization and transport of proteins and RNA. Fundamentally, FUS is a member of Ewing's sarcoma family of proteins that appears to shuttle between the cytoplasm and nucleus.^[^ ^35^ ^]^ Furthermore, according to previous evidence, there are two important pathways involved in FUS's subcellular mislocalizations: Transportin (TRN)‐mediated nuclear import of FUS and arginine methylation‐associated FUS export in HeLa cell culture models.^[^ ^16^, ^36^ ^]^ A study also demonstrated that the K510 site of FUS modified by acetylation could disrupt the interaction between FUS and Transportin‐1, resulting in the localization of FUS in the cytoplasm and the formation of SGs.^[^ ^37^ ^]^ Likewise, FUS contributes to RNA metabolism by exporting RNA to the cytoplasm.^[^ ^38^ ^]^ These results indicate that the subcellular localization of FUS or its function in regulating RNA could play a key role in biological progression. However, the molecular transport and interaction between FUS and circRNAs in breast cancer remain unclear. In our study, we found that FUS could bind to the 3’‐flanking intron region of pre‐TBC1D14 to promote the back splicing of circTBC1D14 and increase the formation of circTBC1D14, was upregulated in TNBC. We found that hypoxia increased the expression of circTBC1D14 and changed its subcellular localization, which might be related to FUS. Previous studies found that PRMT1 could slightly regulate the subcellular localization of FUS in normal conditions,^[^ ^36^ ^]^ but FUS is stress‐associated RBP,^[^ ^39^ ^]^ Then we wondered whether stress could amplify PRMT1‐regulated transferring of FUS–circTBC1D14. Our results demonstrated that arginine methylation‐associated FUS modified by PRMT1 could form a complex with circTBC1D14 and be co‐exported to the cytoplasm under hypoxic conditions. Meanwhile, it was PRMT1 but not FUS that increased the expression of circTBC1D14 under hypoxic conditions. In turn, circTBC1D14 contributes to stabilizing PRMT1 by inhibiting its K48‐associated polyubiquitination. Overall, PRMT1 is a powerful driver of this process and plays a key role in circTBC1D14‐maintained cellular homeostasis under hypoxic conditions.

SGs are triggered by various stress conditions, including heat, osmotic, and oxidative stress.^[^ ^40^ ^]^ SGs are important non‐membrane‐bound cellular constructs that contain high concentrations of protein and RNA. Previous studies have confirmed that most SG components are a subset of RNA‐binding proteins.^[^ ^41^ ^]^ For example, it has been reported that FUS and TDP‐43 granules can be generated in both the nucleus and cytoplasm under weak hyperosmotic stress.^[^ ^42^ ^]^ Furthermore, the formation and dynamics of SGs are devoted to circumventing stress and maintaining homeostasis, which can influence RNA localization, translation, and degradation. It was confirmed that the SG association of long noncoding RNAs was reduced and transported by nuclear import receptors (NIRs), which can equilibrate the localization of SGs. Zhao et al. demonstrated that circVAMP3 interacts with CAPRIN1 to promote SGs formation and inhibit c‐Myc translation in hepatocellular carcinoma.^[^ ^43^ ^]^ All these studies indicate that long noncoding RNAs play a role in the assembly of SGs, which regulate the localization or translation of RNAs. In our study, we found that FUS could interact with circTBC1D14 to generate SGs under hypoxic conditions, which contributed to changes in the subcellular localization of SGs and the maintenance of cancer cell homeostasis in response to stress. Meanwhile, our results indicated that VHL mRNA could co‐localize in FUS‐circTBC1D14 positive‐stress granules, leading to an inhibition of VHL translation, which enhanced HIF‐1*α* overexpression and import to the nucleus.

Autophagy‐regulated clearance of SGs is promoted under cellular stress or disease conditions.^[^ ^44^ ^]^ Autophagy is a regulatory cytoprotective behavior that contributes to the elimination of dysfunctional cellular components and their recycling, balancing the cellular energy needs to respond to stress. Previous studies have reported that a dysregulated interface between autophagy and RBP‐associated SGs may influence the dynamics of SGs.^[^ ^27^, ^45^ ^]^ A complex of C9ORF72 and P62 associated with SGs can be eliminated by autophagy.^[^ ^27a^ ^]^ Our study also found that the levels of hypoxia‐induced FUS–circTBC1D14 SGs were supervised by circTBC1D14 and/or FUS to achieve cellular rebalance. FUS is crucial in the formation of SGs under cellular stress and can interact with P62 at the interface of autophagy and SGs (Figure 2Q–T).^[^ ^27^ ^]^ Additionally, the complex of circTBC1D14 and FUS can enhance Atg5/7/12, P62, and LC3B‐associated autophagy, which is involved in the membrane elongation of autophagic vesicles. Our findings showed that CQ significantly abolished SG‐associated autophagy, indicating that the fusion of autophagosomes and lysosomes is essential for FUS–circTBC1D14‐associated autophagy. We found that circTBC1D14 recruits LAMP1‐positive lysosomes and promotes the interaction between FUS and LAMP1. These findings reveal circTBC1D14 as a potential regulator of stress‐driven autophagy (Figure 7J).

In summary, we identified and characterized an important circRNA, circTBC1D14, which physically interacted with FUS, changed its subcellular localization via PRMT1‐associated FUS transfer, and balanced SG formation and autophagy to maintain cellular homeostasis under hypoxic conditions. These results suggested that circTBC1D14 can be used as a potential diagnostic and therapeutic target in TNBC.

## 4 Experimental Section

### Cell Culture and Transfection

The breast cancer cell lines MDA‐MB‐231(MDA231), MDA‐MB‐468(MDA468), MCF‐7, T47D, and SKBR‐3, the normal breast epithelial cell line MCF‐10A, and the HEK‐293T cell lines were purchased from the American Type Culture Collection (ATCC). T47D cells were cultured in RPMI‐1640 medium (Macgene); SKBR‐3 cells were cultured in McCoy's 5a medium (Macgene); MCF‐10A was propagated in advanced DMEM/F12 medium added with insulin (10 µg mL^−1^), epidermal growth factor (EGF) (20 ng mL^−1^), cholera toxin (100 ng mL^−1^), hydrocortisone (0.5 µg mL^−1^) and 5% horse serum (Gibco); all other cells grew in Dulbecco's modified Eagle's medium (Macgene) as routine. Fetal bovine serum (10% in medium) (Gibco), penicillin (100 U mL^−1^) (Macgene), and streptomycin (100 ng mL^−1^) (Macgene) were supplemented in all media. All cell lines were cultured in a 21% O_2_, 5% CO_2_ incubator at 37 °C. For hypoxic conditions, adherent cells in a regular incubator were transferred to a hypoxic incubator with 1% O_2_ and 5% CO_2_ at 37 °C for a specified gradient of time.

Cells were first transfected with the relative virus reagents for circTBC1D14 knockdown or overexpression. The infected cells were treated with 2.5 µg mL^−1^ puromycin (Sigma–Aldrich) for 2 or 3 days, or with 500 µg mL^−1^ G418 (Sigma–Aldrich) for 5–7 days to obtain stable cells. Following the manufacturer's instructions, Lipofectamine 2000 (Invitrogen) was used in the study to help individual siRNAs (GenePharma) transfect into cells. All RNAi sequences are shown in Table S1 (Supporting Information).

### RT‐PCR and RT‐qPCR

The total RNA was extracted with a TRIzol reagent (Invitrogen). The extracted RNA was then reverse transcribed into cDNA using RT Kit for qPCR (Takara) according to the manufacturer's protocol. The quantitative real‐time PCR (RT‐qPCR) was carried out with the SYBR Green PreMix (Takara) and 1 µg cDNA was used as a template by Light Cycler 480 II Real‐Time PCR System (Roche). The primer sequences in the study were listed in Table S2 (Supporting Information).

### RNase R Treatment and Actinomycin D Assays

For the RNase R treatment assay, the isolated total RNA (2 µg) from MDA231 and MDA468 cells were incubated with or without 3 U µg^−1^ RNase R (Invitrogen) for 30 min at 37 °C. After that, the RNAs were extracted using the TRIzol reagent. Then the reverse transcriptions of the enriched RNA were performed. Circular or linear RNA is detected by PCR analysis using divergent or convergent primers, and then agarose gel electrophoresis. The primer sequences in the study were listed in Table S2 (Supporting Information).

For the actinomycin D assay, the MDA231 and MDA468 breast cancer cells were exposed to 2 µg mL^−1^ actinomycin D (Sigma) or Dimethyl sulfoxide (DMSO) at indicated time point. Then the total RNA was extracted, reverse transcribed, and analyzed by RT‐qPCR. The primer sequences in the study are listed in Table S2 (Supporting Information).

### Cytoplasmic and Nuclear RNA Analysis

Nuclear and cytoplasmic extracts were prepared using the PARIS Nuclear Cytoplasmic Extraction Reagent kit (Invitrogen), following the manufacturer's instructions. Briefly, the pre‐treated cells were digested with trypsin‐EDTA (Macgene), washed with cold phosphate‐buffered saline on ice, and suspended in an ice‐cold cell fractionation buffer. The samples were incubated on ice for 10 min and centrifuged at 4 °C and 500 × *g* for 5 min. The supernatant (cytoplasmic extract) was then transferred to a fresh RNase‐free tube on ice. The insoluble fraction containing nuclei was suspended in an ice‐cold cell disruption buffer and vortexed until the lysate was homogenous. Both cytoplasmic and nuclear extracts were divided into two parts: one was used for RNA isolation with an equal volume of 2 × lysis/binding solution at room temperature, and the other was clarified via centrifugation at 4 °C for 2 min at a high speed to collect the cytoplasmic protein. For RNA isolation, 1.25 volume of 100% ethanol was added to the lysate and mixed. The lysate/ethanol mixture was then passed through a filter cartridge, pre‐placed in a collection tube, and centrifuged for 30 s. The filter cartridge was then washed twice with a working solution mixed with ethanol, followed by centrifugation for 15 s. RNA was eluted twice with preheated (95 °C) nuclease‐free water. RNA and proteins in the nucleus and cytoplasm were subsequently analyzed via RT‐qPCR and western blotting, respectively. Primer sequences used in this study are listed in Table S1 (Supporting Information).

### RNA Immunoprecipitation (RIP)

The RIP assay was carried out with the EZ‐Magna RIP Kit (Millipore). Briefly, the breast cancer cells were lysed using rip lysate on ice for 30 min. Protein A/G magnetic beads were incubated with antibodies to the RNA‐binding proteins (RBPs) for 1 h to produce a magnetic bead antibody complex, the complex was added to the cell suspension and turned over overnight at 4 °C. The RNA bound to the RBP was immobilized using the magnet, while the unbound materials were removed by washing with a washing buffer. The RNAs were purified with the proteinase K buffer and quantitatively analyzed using RT‐qPCR.

### Biotin Probe Synthesis and RNA Pull‐Down Assay

The DNA for circTBC1D14 synthesis was obtained by PCR using T7 promoter sequence fusion primer: sense sequence of forward primer is 5'‐TAATACGACTCACTATAGGGTTTCTCCTTGGACCAAGATGACTGA‐3'; Anti‐sense sequence of forward primer is 5'‐TAATACGACTCACTATAGGGAAAGAGGAACCTGGTTCTACTGACTR‐3'; sequence of the reverse primer is 5'‐CGTGGGTGATATTTAACTCGTTGCC‐3'. RNA was synthesized in vitro using Ambion MAXIscript SP6/T7 Kit (Thermo Fisher) according to the manufacturer's protocols. The RNA complex biotin‐labeled was pulled down by coculturing the cell lysates with streptavidin‐coated magnetic beads (Thermo Fisher) following the manufacturer's instructions from the Pierce Magnetic RNA‐protein Pull‐Down Kit (Thermo Fisher). Briefly, the labeled RNA sequences were firstly co‐incubated with the pre‐washing beads at room temperature for 30 min. The cell lysates were harvested from pretreated breast cells using the standard lysis buffers and were co‐cultured with the RNA‐bonded beads at 4 °C overnight. Then, the mix was washed with an equal volume of 1× wash buffer three times. 50 µL Elution Buffer was added to the beads to isolate RNA‐binding proteins for 30 min at 37 °C. The supernatants were taken, denatured for 10 min at 95 °C, and analyzed by Western blot analysis or mass spectrometry.

### RNA Fluorescence In Situ Hybridization (FISH)

RNA FISH assay was executed using a specific probe synthesized by Genepharma company in the back splice region of circTBC1D14 RNA following the sequence: TTGGTCCAAGGAGAAACGTGGGTGATAT. Then the prehybridization and hybridization experiments were carried out by the Fluorescent in Situ Hybridization Kit (Genepharma) according to the manufacturer's protocol. Briefly, the breast cancer cells pretreated were seeded in glass slides, fixed with 4% paraformaldehyde for 15 min at room temperature, and punched with 0.1% Triton X‐100 for 15 min. After being cultured in 2 × saline sodium citrate (SSC) for 30 min at 37 °C, the hybridization was performed at 37 °C overnight in a dark moist chamber. The next day, 0.1% Tween 20, 2 × SSC, and 1 × SSC were prepared to wash the slides in turn. All detergents are preheated at 42 °C. If immunofluorescence was needed, the slides and Alexa Fluor labeled secondary antibody (Zsbio) were co‐cultured for 2 h at room temperature in a dark moist chamber, and then the slides were washed with 1 × PBS three times. The nucleus was stained with 4, 6‐diamidino‐2‐phenylindole (DAPI). The images were acquired using fluorescence microscopy (ZEISS).

### Immunofluorescence (IF)

The cancer cells were treated as shown in the figures after being seeded in the glass cover slides. The cells were fixed in 4% paraformaldehyde for 20 min at room temperature and then permeabilized with 0.4% Triton X‐100 for 40 min. Subsequently, the cells were sealed with 10% goat serum at room temperature for 1 h. The cells and the primary antibody were incubated in a specific dark moist overnight at 4 °C. After the primary antibody was washed by PBS three times, the cells were incubated with Alexa Fluor labeled secondary antibody without light followed by being dyed with DAPI for 30 min to stain the nucleus. Then the fluorescence signals were captured by a fluorescence microscope (ZEISS).

### Reactive Oxygen Species (ROS)

ROS was detected by using the Reactive Oxygen Species Assay Kit from Sigma. Pretreated breast cancer cells were seeded in 96‐well plates at a density of 15 000 cells per well. After treatment with 3‐MA (3‐Methyladenine) (5 mm) in the hypoxic incubator overnight, the cells culture medium was replaced with a diluted probe (final concentration 10 nm in serum‐free medium) and incubated in a 37 °C hypoxic incubator for 20 min. The cells were washed three times with serum‐free cell culture medium to fully remove the probes. Afterward, fluorescent signals were measured with a multifunctional microplate reader (PerkinElmer) using an excitation wavelength of 488 nm and an emission wavelength of 525 nm.

### JC‐1

The qualitative and quantitative detection of changes in mitochondrial membrane potential during cell death was performed by MitoProbe JC‐1 (Invitrogen). Breast cancer cells (15 000 cells per well) were pretreated with 3‐MA (5 mm) for 24 h, then loaded on 100 µL JC‐1 staining working solution, and incubated at 37 °C for 20 min in the cell culture incubator. Washing twice with JC‐1 staining buffer (1×), the absorbance was measured by a multifunctional microplate reader (PerkinElmer) with excitation light at 490 nm and the emission light at 530 nm for JC‐1 monomer detection, while the excitation light at 525 nm and the emission light at 590 nm for JC‐1 polymer detection. Red fluorescence‐labeled mitochondria with high potentials and green fluorescence‐labeled mitochondria with low potentials, which is an indicator fluorescence‐labeled of apoptosis. The red‐green ratio was calculated as indicated.

### Cytotoxicity Assay

LDH leakage assay was used for cytotoxicity detection according to the instructions by LDH Cytotoxicity Assay Kit (Abcam). After 3‐MA stimulated the drug group for 24 h, the cell was added 150 µL of LDH release reagent instead of the culture supernatant. Continuing to incubate for 1 h, the cell culture plate was then centrifuged at 400 g for 5 min. 120 µL of the supernatant was taken from each well, moved to a new 96‐well plate, mixed with 60 µL of LDH detection working solution, and then incubated at room temperature in the dark for 30 min. For sample detection, dual‐wavelength measurements were performed using a wavelength of 490 nm as a detection wavelength and 600 nm as a reference wavelength. Cytotoxicity (%) = (absorbance of treated sample – absorbance of sample control well) / (absorbance of maximum cell enzyme activity – absorbance of sample control well) × 100.

### Western Blot and Co‐Immunoprecipitation Assays

For Western blot analysis, the cultured cells were collected in a moderate amount of Western and IP lysis buffer (Beyotime) containing 20 mm Tris (pH = 7.5), 150 mm NaCl, 1% Triton x‐100, and 1 mm PMSF. After being centrifuged at 4 °C and 12 000 rpm for 15 min, the lysis product was heated at 95 °C for 10 min with SDS to denature the proteins. Appropriate amounts of proteins were loaded into the SDS‐PAGE gels and then transferred to the PVDF membrane (Millipore). After sealing the non‐specific binding sites on the membrane using 5% skim milk, the primary antibody was incubated overnight at 4 °C. Subsequently, the TBST buffer was then used to wash the membranes three times and horseradish peroxidase (HRP)‐conjugated secondary antibodies specifically bound to the protein labeled by the primary antibody for 2 h. Lastly, the protein blot was developed by enhanced chemiluminescence reagent (Vazyme) and densitometry values were analyzed using ImageJ software. The antibody information used in the study was listed in Table S3 (Supporting Information).

For the co‐Immunoprecipitation (CO‐IP) assay, the cells were lysed by the cell lysis buffer mentioned above. Cell lysates were then divided into the input and IP groups. The IP groups’ protein was incubated with primary antibodies for 2 h followed by the addition of appropriate protein A/G beads (Santa Cruz) overnight at 4 °C. The next day, the beads were washed with IP buffer three times and combined with a secondary antibody for 2 h. Finally, SDS was added to the samples, and a Western blot was used to analyze the immunoprecipitation.

### Cell Proliferation and Clone Formation Assays

The 5‐ethynyl‐2’‐deoxyuridine (EdU) assay was carried out with the EdU proliferation Kit (RiboBio). Briefly, the pretreated cells were digested with trypsin and seeded into 96‐well plates (1 × 10^4^ cells per well). EU solution was prepared with a complete medium and added to the cells for 2 h culturing. The cells were then washed with PBS three times and fixed with 4% paraformaldehyde. The 2 mg mL^−1^ glycine solution was used to neutralize excess fixative fluid. After washing the cells with PBS three times again, 0.5% Triton X‐100 was used to permeate the cells for 10 min. 1× Apollo staining reaction solution and 1× Hoechst reaction solution was prepared and added to the cells for 30 min without light in turn. Immediate observation and photographs were taken with a fluorescence microscope (ZEISS).

For the 3‐(4,5‐dimethylthiazol‐2‐yl)‐2,5‐diphenyltetrazolium bromide (MTT) assay, the pretreatment cells were cultured in a 96‐well plate (1 × 10^4^ per well) and added to 100 µL freshly prepared MTT solution (5 mg mL^−1^ in PBS) in each well for 4 h to allow MTT to be reduced to Formazan. 20 µL dimethyl sulfoxide (DMSO) was added to each well to dissolve Formazan. After shaking gently, the optical density (OD) value was measured with the Microplate Reader (Perkin Elmer).

For colony formation assay, the breast cancer cells were seeded in a 6 cm disk (1 × 10^3^ per well) and incubated for 3 weeks to form colonies. When visible colonies appeared in the dishes, the cells were washed with PBS and fixed with methanol. 2% crystal violet (Sigma) was used to strain for observation.

### Migration and Wound Healing Assays

Migration assays were performed using transwell chambers (Corning) to demonstrate the ability of cells to migrate. Briefly, pretreated breast cancer cells were resuspended in a serum‐free medium and transferred to the chambers by adding 300 µL of the cell suspension to the upper chamber and 700 µL of medium containing 20% ​​fetal bovine serum to the lower chamber. After co‐culturing at 37 °C for 24 h, the migrated cells were fixed with methanol, stained with 2% crystal violet, and counted under a microscope.

For invasion assay, the transwell chambers (Corning) coated with Matrigel (Becton Dickinson) were used to test the invasive capacities of cultured cells. Briefly, Matrigel was pre‐polymerized in transwell chambers at 37 °C for 1 h to reconstitute a basement membrane. 300 µL cell suspension in serum‐free medium was added to the upper chamber, and 700 µL of the medium containing 20% serum was added to the lower chamber. The cells invading the lower surface of the membrane were fixed, stained, and counted as a migration assay.

For the wound healing assay, 2 × 10^5^ cells per well were cultured in a six‐well plate. A straight line was scraped along the diameter of the well with the head of 10 µL pipette tips, and the growth of the cells in the line was observed at 0 and 24 h later.

### Animal Experiments

Six‐week‐old female nude mice were purchased from Jicui Yaokang Biotechnology and housed in the Laboratory Animal Center of Shandong University. To construct the xenograft tumor models, nude mice were randomly divided into four groups, and 1 × 10^7^ MDA231 cells transfected with the control plasmid, circTBC1D14 overexpression vector, circTBC1D14 short hairpin RNA (shRNA), or control shRNA were subcutaneously injected into the mice. Tumor volumes were measured using a caliper every week using the following formula: volume = length × (width/2)^2^. The animals were sacrificed after 35 d, and the tumor tissues were isolated for subsequent weighing and pathological staining.

For the lung metastasis models, nude mice were randomly divided into two groups and injected with 1 × 10^6^ MDA231 cells transfected with the control plasmid and the circTBC1D14 overexpression vector from the tail vein. Animals were sacrificed after 60 d. The lung tissues of the experimental and control groups were separated, weighed, measured, fixed with formalin, dehydrated, and embedded in paraffin for histological analysis. Shandong University Animal Care and Use Committee approved all animal studies described herein(KYLL‐2019(KS)‐113).

### Hematoxylin and Eosin (H&E) and Immunohistochemistry (IHC) Staining

For H&E staining, post‐dehydrated xenograft tissues and lung tissues were embedded in paraffin and cut into slides of 0.4 µm, then the slides were dewaxed in a series of concentration gradient ethanol solutions. Staining was performed with hematoxylin and eosin in turn. For IHC staining, firstly, the paraffin‐embedded tissue sections were deparaffinized as mentioned above, then heated in 10 mm sodium citrate solution (PH = 6) for antigenic repair. After cooling to room temperature, the specific tissue positions were labeled with a histochemical pen. Endogenous peroxidase blocking was performed by 3% H_2_O_2_, followed by goat serum to seal nonspecific binding sites. The primary antibody was incubated at 4 °C overnight. After washing the slides three times with PBS, a biotin‐conjugated secondary antibody from Zhongshan Golden Bridge Biotechnology was used to label the primary antibody for 30 min at room temperature. The slides were stained by diaminobenzidine (DAB), re‐dyed by hematoxylin (Beyotime), and dehydrated through a graded alcohol series. Finally, the slides were sealed with neutral resin and observed under a light microscope (Leica).

### Patients and Samples

This study was approved by the Ethics Review Committee of Qilu Hospital of Shandong University (Jinan, China). All breast cancer and adjacent normal tissues of patients were collected during breast cancer surgeries of patients in Qilu Hospital and stored in an Ultra‐low Temperature Freezer at −80 °C until use. Two pathologists independently assessed all patients and followed up with their prognostic information. Informed consent was obtained from all patients.

### Statistical Analysis

All statistical analysis was calculated by GraphPad Prism 8.0 (GraphPad Software Inc., CA, USA). All results in the figures are shown as mean ± standard deviation (S.D.) in the figures. The Kaplan–Meier survival analysis and the Log‐rank test was used to estimate the Overall Survival (OS). Statistical significance is justified by *p* values using the two‐tailed Student's t‐test, and the levels were set at *p < 0.05, **p < 0.01, ***p < 0.001 and ns means no significance.

## Conflict of Interest

The authors declare no conflict of interest.

## Supporting information

Supporting Information

## Data Availability

The data that support the findings of this study are available from the corresponding author upon reasonable request.
