# Supplementary material for: Hypoxia‐Induced FUS–circTBC1D14 Stress Granules Promote Autophagy in TNBC
Source: Adv Sci (Weinh). 2023 Feb 19;10(10):2204988. doi: 10.1002/advs.202204988 (PMC10074116; doi:10.1002/advs.202204988)
Supplement: Supplementary file 1 — Supporting Information [file ADVS-10-2204988-s001.pdf]

Supplementary Figures and Figure Legends

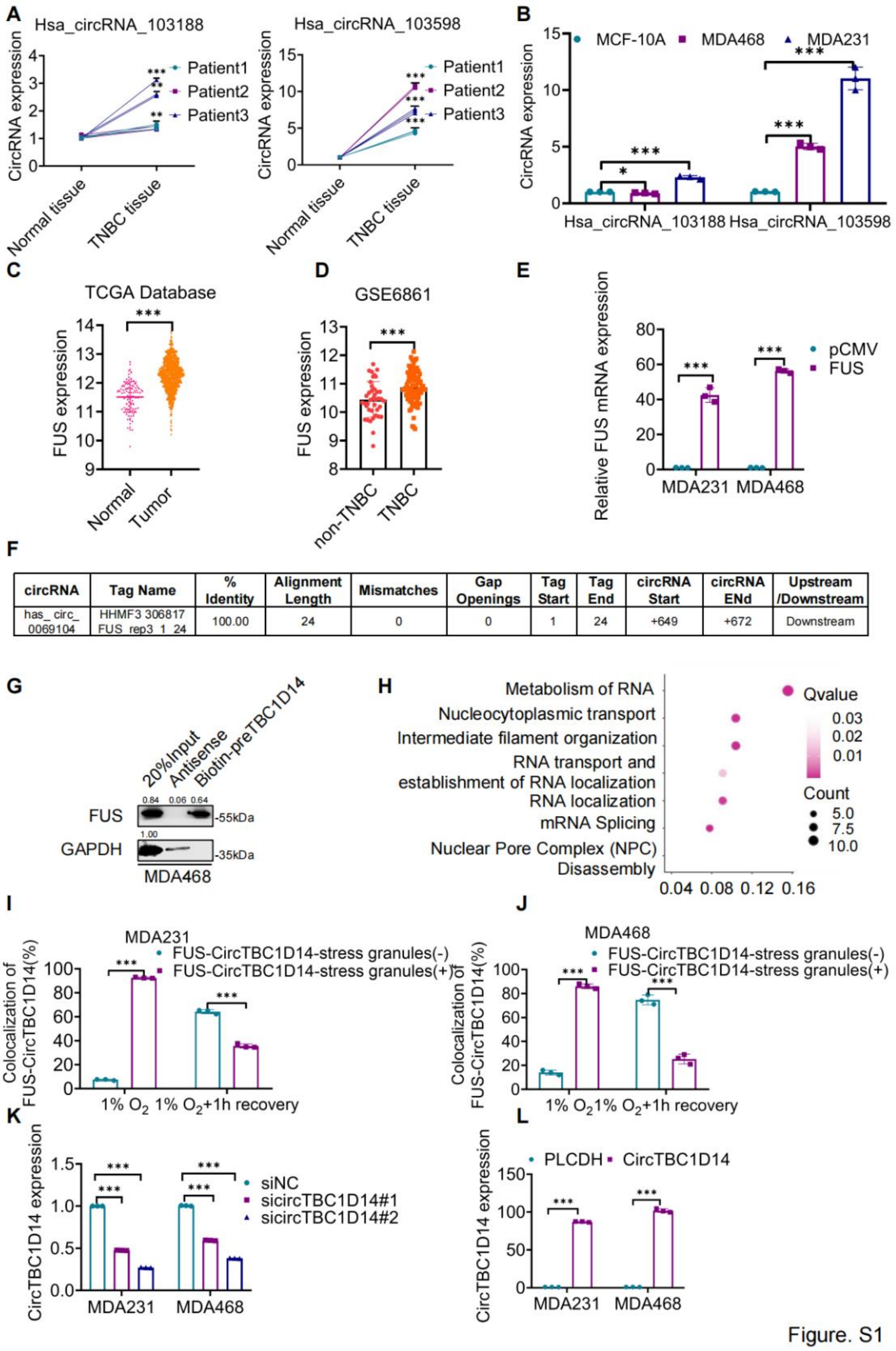

circRNA	Tag Name	% Identity	Alignment Length	Mismatches	Gap Openings	Tag Start	Tag End	circRNA Start	circRNA ENd	Upstream /Downstream
has_ circ_ 0069104	HHMF3 306817 FUS_rep3_1_24	100.00	24	0	0	1	24	+649	+672	Downstream

Figure. S1 FUS was an important regulator of circTBC1D14. A) Quantitative real-time PCR analysis of has_circRNA_103188 and has_circRNA_103598 in breast cancer patient samples. Two-tailed unpaired t-test. B) Quantitative real-time PCR analysis of has_circRNA_103188 and

has_circRNA_103598 in MCF-10A, MDA468, and MDA231 cells. Two-tailed unpaired t-test. C) FUS expression analysis in TCGA database. Two-tailed unpaired t-test. D) FUS expression analysis in the GEO database. Two-tailed unpaired t-test. E) Quantitative real-time PCR analysis of FUS in MDA468 or MDA231 cells with FUS overexpression. Two-tailed unpaired t-test. F) Prediction of the flanking intron downstream of circTBC1D14 binding regions of FUS from online database Circular RNA Interactome. G) Western blot of RNA pull-down assays. H) GO analysis of RNA pulldown mass spectrometry and the related signaling pathways. I and J) Statistical diagram of colocalization percentage of FUS-CircTBC1D14 stress granules in MDA468 and MDA231 cells. Two-tailed unpaired t-test. K and L) Quantitative real-time PCR analysis of circTBC1D14 in MDA468 or MDA231 cells with circTBC1D14 overexpression or knockdown. Two-tailed unpaired t-test. The data are shown as the mean ±SD, NS (no significance) *$P < 0.05$ **$P < 0.01$, ***$P < 0.001$.

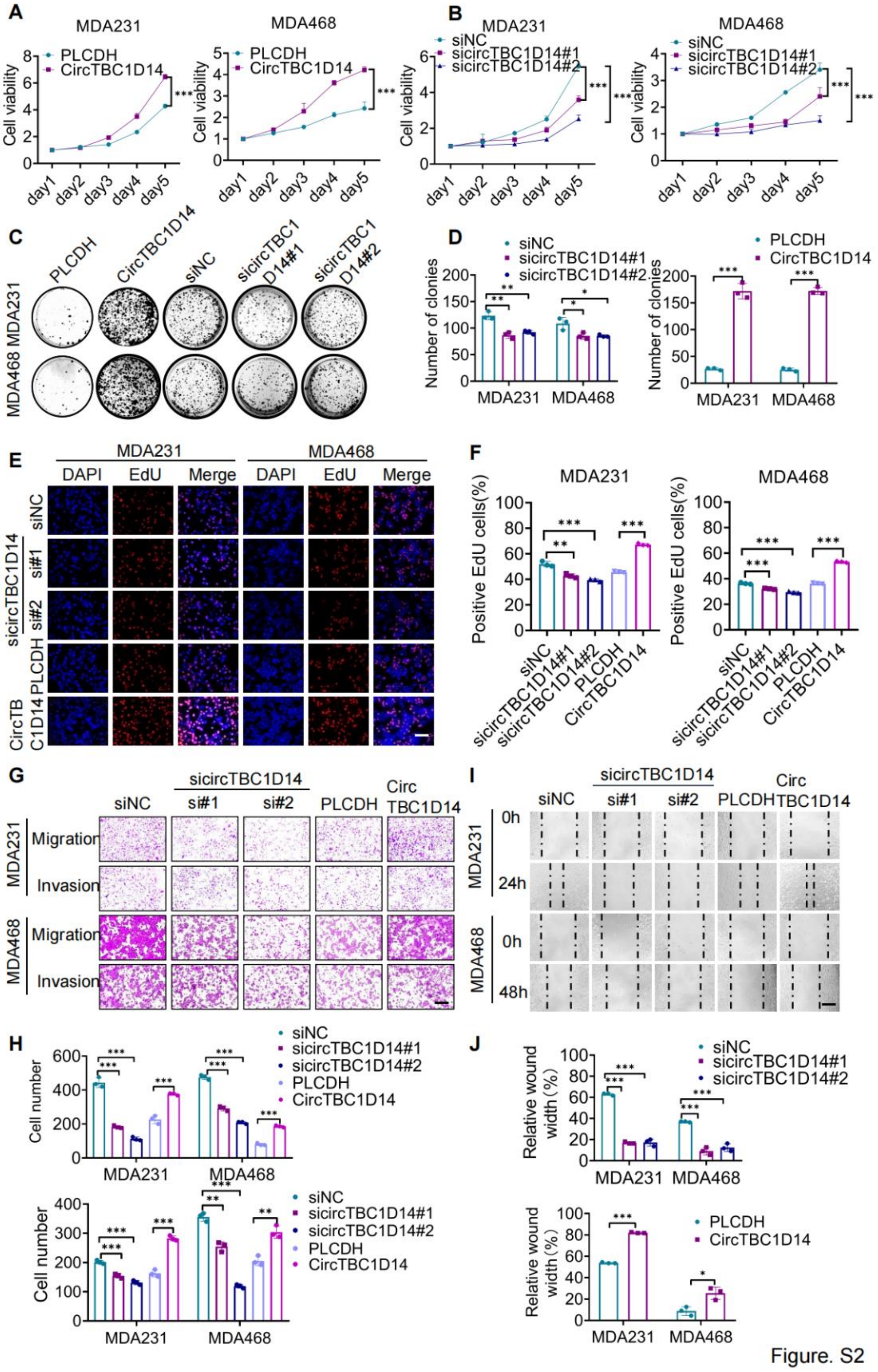

Figure. S2

Figure. S2 CircTBC1D14 was essential for tumor progression and metastasis. A and B) Cell viability of MDA231 and MDA468 cells with circTBC1D14 overexpression or knockdown compared to control cells. Two-tailed unpaired t-test. C) Clone formation of MDA231 and MDA468 cells with circTBC1D14 overexpression or knockdown compared to control cells. D)

Statistical diagram of C). Two-tailed unpaired t-test. E) EdU assay of MDA231 and MDA468 cells with circTBC1D14 overexpression or knockdown compared to control cells. Scale bar, 20 µm. F) Statistical diagram of E). Two-tailed unpaired t-test. G) Transwell assay of MDA231 and MDA468 cells with circTBC1D14 overexpression or knockdown compared to control cells. Scale bar, 20 µm. H) Statistical diagram of G). Two-tailed unpaired t-test. I) Wound healing assay of MDA231 and MDA468 cells with circTBC1D14 overexpression or knockdown compared to control cells. Scale bar, 20 µm. J) Statistical diagram of I). Two-tailed unpaired t-test. The data are shown as the mean ±SD, NS (no significance) **P* < 0.05 ***P* < 0.01, ****P* < 0.001.

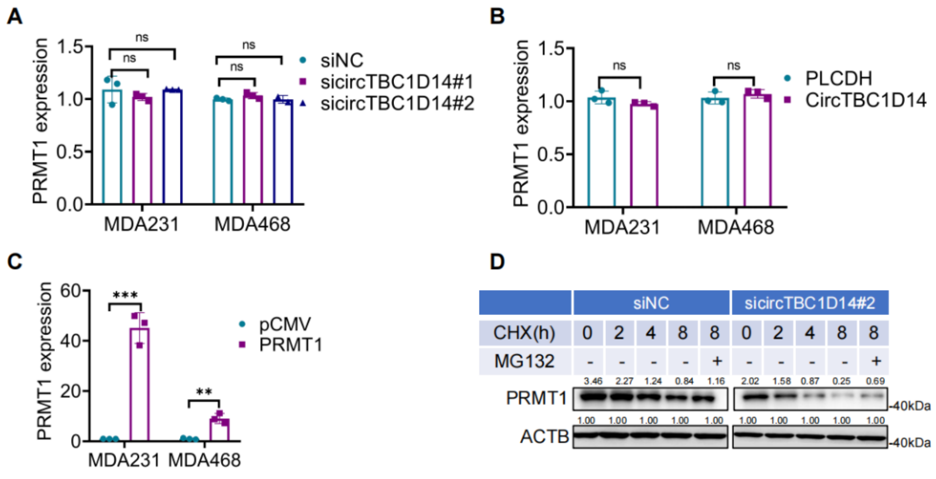

Figure. S3

Figure. S3 Hypoxia-induced interaction between circTBC1D14 and PRMT1. A and B) Quantitative real-time PCR analysis of PRMT1 expression in MDA468 or MDA231 cells with circTBC1D14 overexpression or knockdown. Two-tailed unpaired t-test. C) Quantitative real-time PCR analysis of RNA expression in MDA468 or MDA231 cells with PRMT1 overexpression. Two-tailed unpaired t-test. D) Western blot of PRMT1 with MG132 treatments pre-treated with CHX in time gradient. The data are shown as the mean ±SD, NS (no significance) **P* < 0.05 ***P* < 0.01, ****P* < 0.001.

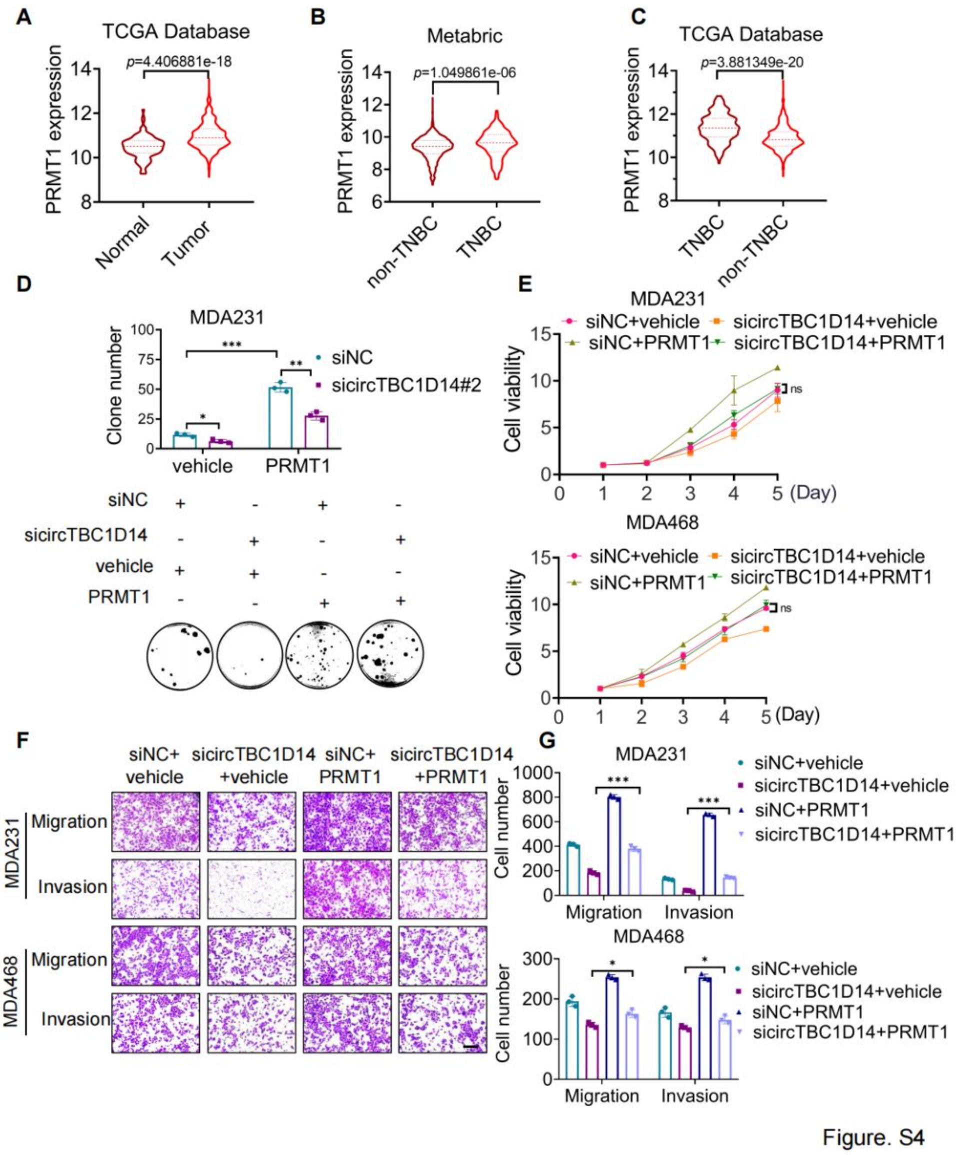

Figure. S4 CircTBC1D14 promoted tumor growth and metastasis by regulating PRMT1 in TNBC. A and C) PRMT1 expression analysis in TCGA database. Two-tailed unpaired t-test. B)

PRMT1 expression analysis in Metabric database. Two-tailed unpaired t-test. D) Clone formation of circTBC1D14 knockdown in MDA231 cells with PRMT1 overexpressed. A statistic diagram was shown in the upper region. Two-tailed unpaired t-test. E) Cell viability of circTBC1D14 knockdown in MDA231 and MDA468 cells with PRMT1 overexpressed. Two-tailed unpaired t-test. F) Transwell of circTBC1D14 knockdown in MDA231 and MDA468 cells with PRMT1 overexpressed. G) Statistical diagram of F). Scale bar, 20 μm. Two-tailed unpaired t-test. The data are shown as the mean ±SD, NS (no significance) *$P < 0.05$ **$P < 0.01$, ***$P < 0.001$.

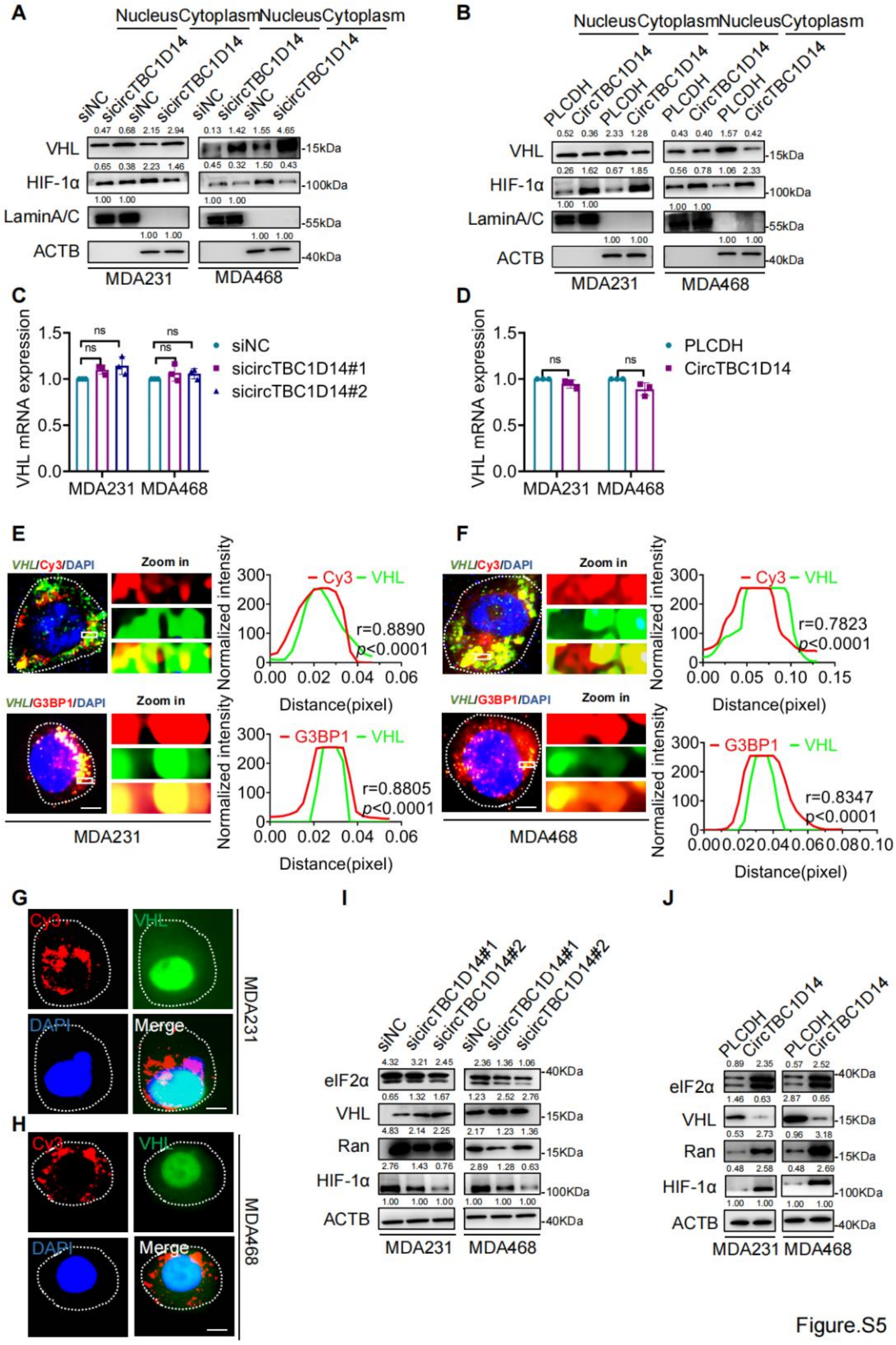

Figure. S5 CircTBC1D14 stabilized hypoxia-inducible factor-1-alpha (HIF-1a) expression by inhibiting the translation of von Hippel-Lindau (VHL). A and B) Western blot analysis of relative distribution with circTBC1D14 overexpression or knockdown in MDA231 and MDA468 cells. C and D) Quantitative real-time PCR analysis of VHL expression in MDA231 or MDA468

cells with circTBC1D14 overexpression or knockdown condition. Two-tailed unpaired t-test. E and F) RNA FISH of VHL and circTBC1D14 or SGs (G3BP1+) in MDA231 and MDA468 cells. Scale bar, 10 μm. Right, Pearson correlation analysis of VHL and circTBC1D14 or SGs (G3BP1+) co-localizing. G and H) FISH of circTBC1D14 and VHL protein in MDA231 and MDA468 cells. Scale bar, 10 μm. I and J) Western blot analysis of relative expression with circTBC1D14 overexpression or knockdown in MDA231 and MDA468 cells. The data are shown as the mean ±SD, NS (no significance) *$P < 0.05$ **$P < 0.01$, ***$P < 0.001$.

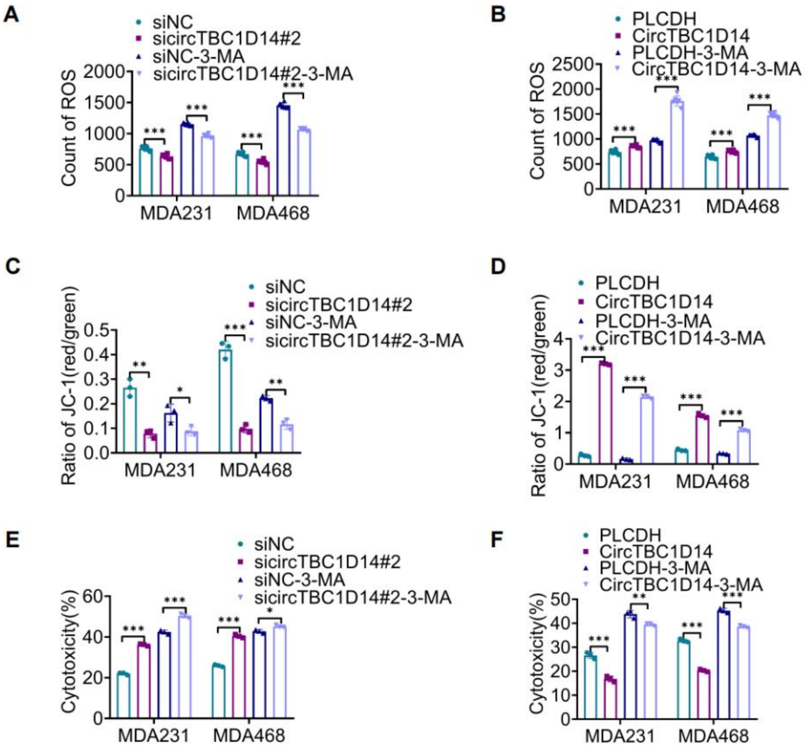

Figure. S6

Figure. S6 CircTBC1D14 regulated cell death via autophagy in hypoxic conditions. A and B) Quantification of the count of ROS with circTBC1D14 overexpression or knockdown in MDA231 and MDA468 cells after 3-MA (5mM) and hypoxia treatment. Two-tailed unpaired t-test. C and D) Quantification of the ratio of JC-1 with circTBC1D14 overexpression or knockdown in MDA231 and MDA468 cells after 3-MA (5mM) and hypoxia treatment. Two-tailed unpaired t-test. E and F) Quantification of the percentage of cytotoxicity with circTBC1D14 overexpression or knockdown in MDA231 and MDA468 cells after 3-MA (5mM) and hypoxia treatment. Two-tailed unpaired

t-test. The data are shown as the mean ±SD, NS (no significance) *P < 0.05 **P < 0.01, ***P < 0.001.

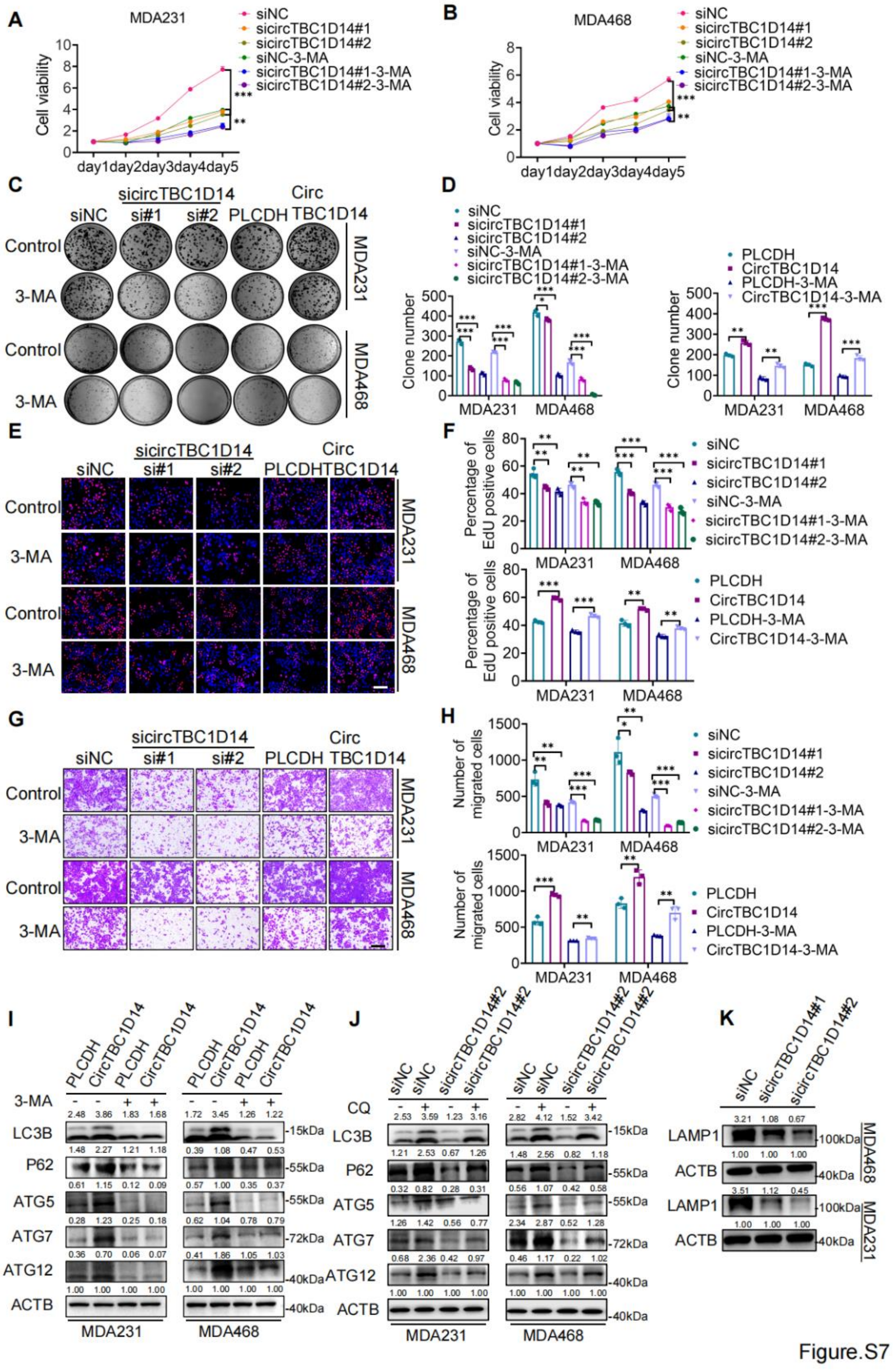

Figure. S7 CircTBC1D14 promoted TNBC proliferation and metastasis through autophagy. A and B) Cell viability of MDA231 and MDA468 cells with circTBC1D14 overexpression or

knockdown compared to control cells after 3-MA (5mM) treatment. Two-tailed unpaired t-test. C) Clone formation of MDA231 and MDA468 cells with circTBC1D14 overexpression or knockdown compared to control cells after 3-MA (5mM) treatment. D) Statistical diagram of C). Two-tailed unpaired t-test. E) EdU assay of MDA231 and MDA468 cells with circTBC1D14 overexpression or knockdown compared to control cells after 3-MA (5mM) treatment. Scale bar, 20 μm. F) Statistical diagram of E). Two-tailed unpaired t-test. G) Transwell assay of MDA231 and MDA468 cells with circTBC1D14 overexpression or knockdown compared to control cells after 3-MA (5mM) treatment. Scale bar, 20 μm. H) Statistical diagram of G). Two-tailed unpaired t-test. I) Western blot analysis of relative expression with circTBC1D14 overexpression after 3-MA (5mM) treatment or not in MDA231 and MDA468 cells. J) Western blot analysis of relative expression with circTBC1D14 knockdown after CQ (25 μM) treatment or not in MDA231 and MDA468 cells. K) Western blot analysis of relative expression with circTBC1D14 knockdown in MDA231 and MDA468 cells. The data are shown as the mean ±SD, NS (no significance) $^{*}P < 0.05$ $^{**}P < 0.01$, $^{***}P < 0.001$.

Supplementary Tables

Table S1 Sequences of siRNA used in this study

Definition	sequences
siCircTBC1D14#1	5'-UAAAUAUCACCCACGUUUCTT-3'
siCircTBC1D14#2	5'-CACCCACGUUUCUCCUUGGTT-3'
siNC	5'-UUCUCCGAACGUGUCACGUTT-3'
siFUS#1	5'-CAGAGUUACAGUGGUUAUA-3'
siFUS#2	5'-CCAAUUCCUGAUCACCCAA-3'
siPRMT1#1	5’-GCGAGGAGATCTTCGGCACCA-3’
siPRMT1#2	5’-GGACATGACATCCAAAGAT-3’
siLAMP1	5’-AGAAAUGCAACACGUUAUU-3’

Table S2 Sequences of primer used in this study

Gene	Primer sequences
GAPDH	F: 5'-GTCTCCTCTGACTTCAACAGCG-3'
	R: 5'-ACCACCCTGTTGCTGTAGCCAA-3'
U6	F: 5'-CTCGCTTCGGCAGCACAT-3'
	R: 5'-TTTGCGTGTCATCCTTGCG-3'
CircTBC1D14	F: 5'-GCTTAGCCATTGGCAACGAG-3'
	R: 5'-GAAGGGGGTTTCCTGGTCG-3'
LinearTBC1D14	F: 5'-GACCAGGAAACCCCCTTCAG-3'
	R: 5'-AGGGTAGGAATCCCCGAGTC-3'
ATCB	F: 5'-CACCATTGGCAATGAGCGGTTC-3'
	R: 5'-AGGTCTTTGCGGATGTCCACGT-3'
FUS	F: 5'-CAAGGCCTGGGTGAGAATGT-3'
	R: 5'-TTGCCTCTCCCTTCAGCTTG-3'
TBC1D14	F: 5'-GAAACGCTGTGCTCACCTGGAA-3'
	R: 5'-GCTCCAGACTTTGCCTCTCACA-3'
PRMT1	F: 5'-TGCGGTGAAGATCGTCAAAGCC-3'
	R: 5'-GGACTCGTAGAAGAGGCAGTAG-3'
LAMP1	F: 5'-CGTGTCACGAAGGCGTTTTCAG-3'
	R: 5'- CTGTTCTCGTCCAGCAGACACT-3'

Table S3 List of antibodies used in this study

Antibody	Catalog Number	Company
Anti-human ATG5 Rabbit Polyclonal Antibody	10181-2-AP	Proteintech Group, Inc
Anti-human ATG7 Rabbit Polyclonal Antibody	10088-2-AP	Proteintech Group, Inc
Anti-human ATG12 Rabbit Polyclonal Antibody	11122-1-AP	Proteintech Group, Inc
Anti-human LC3 Rabbit Polyclonal Antibody	14600-1-AP	Proteintech Group, Inc
Anti-human GAPDH Rabbit Polyclonal Antibody	10494-1-AP	Proteintech Group, Inc
Anti-human Lamin A/C Rabbit Polyclonal Antibody	10298-1-AP	Proteintech Group, Inc
Anti-human Beta Actin Mouse Monoclonal Antibody	66009-1-Ig	Proteintech Group, Inc
Anti-human MYC-Tag Mouse Monoclonal antibody	60003-2-Ig	Proteintech Group, Inc
Anti-human Flag-Tag Rabbit Polyclonal antibody	20543-1-AP	Proteintech Group, Inc
Anti-human HA-Tag Mouse Monoclonal Antibody	66006-2-Ig	Proteintech Group, Inc
Anti-human FUS Rabbit Polyclonal Antibody	11570-1-AP	Proteintech Group, Inc
Anti-human PRMT1 Rabbit Polyclonal Antibody	11279-1-AP	Proteintech Group, Inc
Anti-human LAMP1 Rabbit Polyclonal Antibody	21997-1-AP	Proteintech Group, Inc
Anti-human KI67 Rabbit Polyclonal antibody	27309-1-AP	Proteintech Group, Inc
Anti-human G3BP1 Rabbit Polyclonal antibody	13057-2-AP	Proteintech Group, Inc
Anti-human P62 Mouse Monoclonal Antibody	sc-28359	Santa Cruz Biotechnology
Anti-human VHL Mouse Monoclonal Antibody	sc-135657	Santa Cruz Biotechnology
Anti-human Ran Mouse Monoclonal Antibody	sc-271376	Santa Cruz Biotechnology
Anti-human eIF4AIII Mouse Monoclonal Antibody	sc-365549	Santa Cruz Biotechnology
Anti-human PARP1 Mouse Monoclonal antibody	sc-8007	Santa Cruz Biotechnology
Anti-human FMRP Rabbit Monoclonal Antibody	4317	Cell Signaling Technology
Anti-human HIF-1α Rabbit Monoclonal Antibody	36169	Cell Signaling Technology
Goat anti-Rabbit IgG (H+L) Highly Cross-Adsorbed Secondary Antibody, Alexa Fluor® 488	ZF-0511	Zhong Shan Golden Bridge Biotechnology
Goat anti-Rabbit IgG (H+L) Cross-Adsorbed Secondary Antibody, TRITC	ZF-0317	Zhong Shan Golden Bridge Biotechnology
Goat anti-Mouse IgG (H+L) Highly Cross-Adsorbed Secondary Antibody, Alexa Fluor® 488	ZF-0513	Zhong Shan Golden Bridge Biotechnology
